# Protein Substitute Requirements of Patients with Phenylketonuria on BH4 Treatment: A Systematic Review and Meta-Analysis

**DOI:** 10.3390/nu13031040

**Published:** 2021-03-23

**Authors:** Fatma Ilgaz, Cyril Marsaux, Alex Pinto, Rani Singh, Carmen Rohde, Erdem Karabulut, Hülya Gökmen-Özel, Mirjam Kuhn, Anita MacDonald

**Affiliations:** 1 Department of Nutrition and Dietetics, Faculty of Health Sciences, Hacettepe University, 06100 Ankara, Turkey; fatma.celik@hacettepe.edu.tr (F.I.); hgokmen@hacettepe.edu.tr (H.G.-Ö.); 2 Danone Nutricia Research, 3584CT Utrecht, The Netherlands; mirjam.kuhn@danone.com; 3 Department of Dietetics, Birmingham Women’s and Children’s Hospital, Birmingham B4 6NH, UK; alex.pinto@nhs.net (A.P.); Anita.Macdonald@nhs.net (A.M.); 4 Metabolic Genetics Nutrition Program, Department of Human Genetics, Emory University, Atlanta, GA 30322, USA; rsingh@emory.edu; 5 Department of Paediatrics of the University Clinics Leipzig, University of Leipzig, 04103 Leipzig, Germany; Carmen.Rohde@medizin.uni-leipzig.de; 6 Department of Biostatistics, Faculty of Medicine, Hacettepe University, 06100 Ankara, Turkey; ekarabul@hacettepe.edu.tr

**Keywords:** phenylalanine hydroxylase deficiency, hyperphenylalaninemia, PKU, protein substitute, medical formula, amino acid mixture, tetrahydrobiopterin, sapropterin, BH4

## Abstract

The traditional treatment for phenylketonuria (PKU) is a phenylalanine (Phe)-restricted diet, supplemented with a Phe-free/low-Phe protein substitute. Pharmaceutical treatment with synthetic tetrahydrobiopterin (BH4), an enzyme cofactor, allows a patient subgroup to relax their diet. However, dietary protocols guiding the adjustments of protein equivalent intake from protein substitute with BH4 treatment are lacking. We systematically reviewed protein substitute usage with long-term BH4 therapy. Electronic databases were searched for articles published between January 2000 and March 2020. Eighteen studies (306 PKU patients) were eligible. Meta-analyses demonstrated a significant increase in Phe and natural protein intakes and a significant decrease in protein equivalent intake from protein substitute with cofactor therapy. Protein substitute could be discontinued in 51% of responsive patients, but was still required in 49%, despite improvement in Phe tolerance. Normal growth was maintained, but micronutrient deficiency was observed with BH4 treatment. A systematic protocol to increase natural protein intake while reducing protein substitute dose should be followed to ensure protein and micronutrient requirements are met and sustained. We propose recommendations to guide healthcare professionals when adjusting dietary prescriptions of PKU patients on BH4. Studies investigating new therapeutic options in PKU should systematically collect data on protein substitute and natural protein intakes, as well as other nutritional factors.

## 1. Introduction

Phenylketonuria (PKU) is an inborn error of phenylalanine (Phe) metabolism caused by deficiency of the Phe hydroxylase enzyme (PAH; EC 1.14.16.1), which catalyzes the conversion of Phe to tyrosine, with the help of the cofactor tetrahydrobiopterin (BH4) [1]. PKU is a rare disorder affecting approximately 1 in 24,000 newborns globally [2], although incidence varies greatly across ethnicities and geographic regions. Infants are usually diagnosed via newborn screening in the first 2 weeks of life and commence treatment if blood Phe levels exceed 360 μmol/L [3]. Untreated, PKU may cause severe neurological impairment with profound intellectual disability [1,3,4]. 

The traditional treatment for PKU is a Phe-restricted diet, which aims to avoid excessive accumulation of Phe to prevent adverse neurocognitive and psychological outcomes, while also meeting requirements for growth and development [3,5,6]. Phe tolerance, the maximum amount that can be eaten whilst maintaining blood Phe levels in the therapeutic range, varies between patients; it is influenced by the residual PAH activity and therefore the severity of PKU [3], and up to 80% of patients tolerate less than 10 g/day natural protein [7]. Therefore, a low-Phe diet requires supplementation with a Phe-free or low-Phe protein substitute, i.e., a protein replacement formula, based on either free L-amino acids (AA), or casein glycomacropeptide (cGMP) supplemented with free AA. Most protein substitutes contain additional tyrosine, micronutrients, essential fatty acids, and long-chain polyunsaturated fatty acids [6]. Protein substitutes are not only necessary to meet age-appropriate protein requirements for growth and to provide tyrosine [3,6], they also improve Phe tolerance and optimize metabolic control by suppressing blood Phe levels [6,8,9,10]. This is particularly important during illness and trauma, where protein substitutes have a protective role by counter-acting protein catabolism [6].

Although successful, dietary treatment of PKU constitutes a substantial burden for patients and their families. The difficulties to adhere life-long to this restrictive diet, as well as to maintain blood Phe levels within the recommended range, have called for new therapies to improve patients’ quality of life [11]. Over the last 12 years, pharmaceutical adjunct therapies have been licensed including treatment with sapropterin dihydrochloride (a synthetic form of BH4) [12] and enzyme substitution therapy with pegvaliase (pegylated recombinant Phe ammonia lyase, PEG-PAL) [13]. Sapropterin therapy is prescribed to BH4-responsive patients with PKU; pegvaliase is only licensed for adults (≥16 y in Europe) with blood Phe levels ≥600 µmol/L. Both pharmaceutical treatments may be used as monotherapies or in combination with Phe restriction. Kure et al. were among the first to report that oral administration of BH4 to some individuals with mild hyperphenylalaninemia led to a significant reduction in blood Phe levels [14]. Since then, it has been suggested that 20–50% of patients with PKU respond to sapropterin [15,16,17,18,19]. The basis of responsiveness may be associated with different molecular mechanisms. Increased liver BH4 concentrations may stimulate the activity of a partially active mutant PAH enzyme [20], as some mutations can decrease the enzyme affinity for its cofactor [21,22], or may act as a chemical chaperone to stabilize mutant PAH [22,23]. Potential responsiveness to BH4 may be predicted from a patient’s *PAH* genotype and/or BH4 loading tests [3,24,25,26]. It varies according to metabolic phenotype—milder forms of PKU are more likely to respond, whereas patients with classic PKU are less likely to do so [2]. 

In responders, the BH4-induced decrease in blood Phe concentrations usually enables an increase in Phe/natural protein tolerance and, thereby, some relaxation of the Phe-restricted diet with lowering or cessation of protein substitute use. However, Phe tolerance is also affected by other factors including severity of PKU, patient’s age, dosage of protein substitute, growth rate, and target blood Phe concentrations [3,27]. Additionally, it has been shown that some adolescents and young adults with PKU are able to tolerate more natural protein than prescribed when challenged [28]. This supports a periodic re-evaluation of Phe tolerance in all patients including responders to BH4 therapy. 

The ultimate goals of BH4 treatment are to (1) allow dietary Phe relaxation and (2) obtain good metabolic control. If either objective is not achieved and sustained long term, continuation of BH4 treatment should be reconsidered. Protein substitutes are a major supplier of nutrients, not only of protein, but also of vitamins and minerals, leading to concerns about the impact on nutritional status of patients taking BH4 when they are stopped [29,30]. This highlights the importance of a systematic and gradual approach when considering reduction of protein substitute, while maximizing natural protein intake in patients on BH4 treatment, in order to avoid impairment of metabolic control and maintain nutritional status. To date, few dietary protocols are available to guide such adjustments [31].

Therefore, the present systematic review aimed to investigate the usage of protein substitute with BH4 therapy and to define criteria for continued protein substitute administration with BH4.

## 2. Methods

### 2.1. Terminology

In this manuscript, “BH4” refers to both the earlier synthetic BH4 formulation (6R-BH4; Schircks Laboratories) (mainly used in studies before 2009) and the later formulation sapropterin dihydrochloride (Kuvan^®^; Merck Serono or BioMarin Pharmaceutical Inc.). “Protein substitute” refers to the Phe-free/low-Phe protein replacement formula. Other names for protein substitute include synthetic protein, amino acid mixture (AAM), AA supplement, casein glycomacropeptide (cGMP or GMP-AA), and (special) medical food/formula. In contrast, we use “natural protein” as a synonym for intact protein.

### 2.2. Literature Search

Using the ProQuest platform, we performed a systematic literature search in a total of 92 electronic databases (including Medline, Embase, SciSearch and BIOSIS Previews) for any articles published in English between 1 January 2000 and 2 March 2020. The full list of electronic databases searched can be found here: https://dialog.com/commercial-databases/, accessed on 2 March 2020. The year 2000 was chosen as the starting date because responsiveness to BH4 was first reported by Kure and colleagues in 1999 [14] and BH4 was not used in PKU management until later. The following search string was used: Ti,ab((“phenyl ketonuri*” OR phenylketonuri* OR PKU OR “phenylalanine deficiency” OR “phenylalanine hydroxylase deficiency” OR “PAH deficiency” OR hyperphenylalaninemia OR hyperphenylalaninaemia OR HPA) AND (biopterin OR BH4 OR thb OR tetrahydrobiopterin OR sapropterin OR kuvan* OR biopten*)).

The Embase database includes many conference abstracts. At the time of the search, Embase covered the International Congress of Inborn Errors of Metabolism (ICIEM) as well as the annual symposia of the Society for the Study of Inborn Errors of Metabolism (SSIEM) and the annual meetings of the Society for Inherited Metabolic Disorders (SIMD) from 2009 until 2018. Therefore, in addition to the database search, electronic copies of the abstract books were retrieved and screened manually for SIMD 2019 and SSIEM 2019.

The Preferred Reporting Items for Systematic Reviews and Meta-Analyses (PRISMA) guidelines [32] were followed and the protocol published on the PROSPERO international prospective register of systematic reviews (CRD42020177311; www.crd.york.ac.uk/PROSPERO, accessed on 30 September 2020).

### 2.3. Study Selection

The PICO (population, intervention, comparison, outcomes) method was applied to formulate the review question, as well as to determine the eligibility criteria. All retrospective and prospective longitudinal studies, randomized controlled trials, and cross-sectional and case–control studies conducted in patients with hyperphenylalaninemia or PKU were included. Conference abstracts were also considered. To be eligible, studies had to include a minimum of 5 long-term BH4-responsive patients (no age restriction and no restriction regarding methodology for assessment of BH4 responsiveness), first treated with a Phe-restricted diet and protein substitute (PS), and subsequently being treated with BH4 for a minimum of 3 consecutive months from first dose received. Preclinical studies (in vitro and in vivo studies conducted on cell cultures or animals), case reports (with <5 BH4 responders), theses, non-original research (such as expert opinions and reviews), and studies without any information on protein substitute use were excluded. Patients with a diagnosis of BH4 deficiency or maternal PKU, or who were treated with pegvaliase were excluded. Patients who had never been prescribed protein substitute, who were treated with BH4 for <3 months, who had been found to be long-term non-responders, or who were not adherent with their treatment were removed from the analyses when known. 

Two reviewers (F.I. and C.M.) screened titles and abstracts independently according to eligibility criteria. The full texts of all potentially relevant articles were reviewed. Conference abstracts without full text were kept if they (or the associated poster when available) contained sufficient information on the primary outcomes. Disagreements were resolved through discussion with all authors.

### 2.4. Outcome Measures

The primary outcomes were prescribed or self-reported intakes of protein substitute, natural protein, total protein, and dietary Phe. Secondary outcomes were nutritional status (i.e., micronutrient and fatty acid blood concentrations or dietary intakes), growth, metabolic control (e.g., blood Phe levels), authors’ definition of protein adequacy (e.g., Food and Agriculture Organization of the United Nations/World Health Organization/United Nations University (FAO/WHO/UNU) safe levels of protein intake or national recommendations/reference amounts for protein intake), and authors’ protocols for change in protein substitute prescription with BH4.

### 2.5. Data Extraction

Data were collected by 2 independent authors (F.I. and C.M.) using a standardized data extraction form and were checked by a third author (A.P.). Information extracted was (1) study characteristics (authors, publication year, country, and design of the study), (2) description of population (sample size and number of BH4 responders, methodology for assessment of BH4 responsiveness, gender, age, type of HPA/PKU, and ethnicity), (3) description of BH4 treatment (time of initiation, dose, drug type, duration, and adherence), (4) primary outcomes (intakes, before BH4 treatment and at follow-up, of protein substitute, natural and total protein, special low-protein foods, any additional supplements, and Phe tolerance), and (5) secondary outcomes (authors’ protocols for natural protein and protein substitute prescriptions, nutritional status, growth, and blood Phe control). Authors of papers where relevant information was missing or ambiguous were contacted to obtain further information/clarification.

### 2.6. Quality Appraisal and Risk of Bias Assessment

Two reviewers (F.I. and C.M.) independently assessed the quality of the evidence and the risk of bias of the included studies using the “Quality Assessment Tool for Before-After (Pre-Post) Studies with No Control Group” [33]. This tool was developed jointly by the U.S. National Heart, Lung and Blood Institute (NHLBI, National Institutes of Health) and Research Triangle Institute (RTI) International. It includes 12 items to evaluate potential flaws in study methods or implementation, including sources of bias (e.g., patient selection, performance, attrition, and detection), confounding, study power, the strength of causality in the association between interventions and outcomes, as well as other factors. Each item was rated as “yes”, “no”, “cannot determine”, “not reported”, or “not applicable”. Based on the ratings, we made an overall judgement regarding the quality of each study: (1) “good quality” if the study had minimal risk of bias, (2) “fair quality” if the study was susceptible to some bias but not deemed sufficient to invalidate its results, and (3) “poor quality” if the study raised substantial concerns. Differing ratings between reviewers were discussed until consensus was reached.

### 2.7. Data Analysis

The analyses considered long-term BH4 responders (as defined by the authors, and, if individual data were available, by considering both the long-term increase in Phe intake and the long-term decrease in blood Phe levels), who had need for a protein substitute before BH4 therapy, and who had been treated with BH4 for at least 3 months to ensure any changes in outcomes were reliable.

For the main outcomes, meta-analyses were performed to compare means before and after start of BH4 therapy, if a minimum of 2 studies were available. Heterogeneity between studies was estimated using the I^2^ statistic, with values of 25%, 50%, and 75% considered to indicate low, medium, and high heterogeneity, respectively. Given the heterogeneity level between studies, we used a random-effects model to calculate pooled estimates with the “metafor 2.4-0” package of R software version 4.0.3 (R foundation for statistical computer, Vienna, Austria) [34]. Because of the relatively small number of studies, we preferred to calculate the 95% confidence intervals using a t-distribution (with degrees of freedom = number of studies-1). As studies reported dietary Phe and protein intakes using different scales (e.g., mg/kg/day or mg/day for Phe intake), the standardized mean difference (SMD) was used to standardize the results to the same scale (SMD = mean change/standard deviation of change). Mean change was obtained by subtracting the mean at follow-up from the baseline mean. However, this method cannot be used to determine the standard deviation of changes because it is not known whether the changes were consistent or variable across individuals. Hence, the standard deviations for the changes were calculated by using 1 of the 2 following methods: (1) the original baseline and final follow-up measurements if individual data were available, or (2) statistical analyses comparing the changes (e.g., confidence intervals, t-values, or *p*-values) if they were presented in the original articles [35]. For the meta-analyses, a *p*-value less than 0.05 was considered statistically significant. Sensitivity analyses considering only studies reporting outcomes in the same unit were also performed. Furthermore, when heterogeneity was particularly high (e.g., I^2^ > 95%), additional sensitivity analyses considered the exclusion of studies that were suspected to contribute most to the heterogeneity. Studies that could not be included in the meta-analyses (i.e., insufficient data or results not reported as means and SDs but only as medians and interquartile range) were analyzed qualitatively. Secondary outcomes were analyzed qualitatively (no meta-analysis).

All data analyzed (both quantitatively and qualitatively) are discussed and used to derive recommendations for which data should be reported at a minimum when investigating responsiveness of PKU patients to BH4; the being aim to improve future data reporting.

## 3. Results

### 3.1. Study Selection

**Figure 1:**
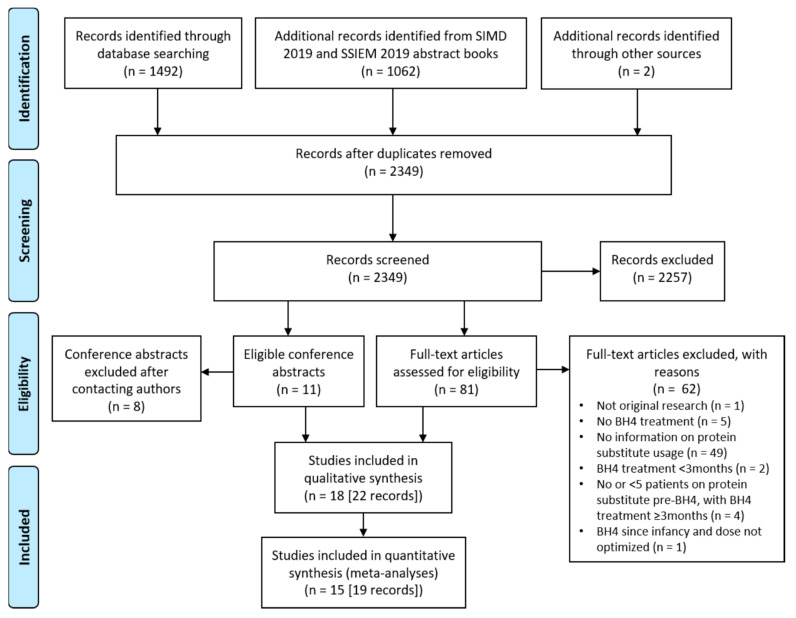
Study selection process according to the Preferred Reporting Items for Systematic Reviews and Meta-Analysis (PRISMA) flow chart.

Of 2349 unique published articles and conference abstracts identified, 19 eligible articles [16,17,29,36,37,38,39,40,41,42,43,44,45,46,47,48,49,50,51] and 3 conference abstracts [52,53,54], describing a total of 18 studies, were included in the systematic review (Figure 1). Three articles [39,42,43] reported data for the same study first published by Singh et al. (2011) [48], and 1 conference abstract [52] reported additional data for the same study published by Ünal et al. (2015) [50]. All 4 were included in the systematic review. Out of a total of 18 studies, 15 were included in the meta-analyses (pre-/post-BH4 data were lacking for two studies [51,53], and only medians were provided in Aldámiz et al. [36]).

### 3.2. Study Characteristics

The characteristics of the 18 studies included are summarized in Table 1. These studies described a total of 306 PKU patients with long-term use of BH4. Most studies were longitudinal (retrospective or prospective) and conducted in Europe (Europe, *n* = 14; USA, *n* = 3; and Turkey, *n* = 1). Sample size varied from 6 to 51, after excluding some patients from the original dataset who did not meet our inclusion criteria (i.e., long-term responders treated with BH4 for ≥3 months and who had been on a Phe-restricted diet and protein substitute(s) before BH4). Different protocols were used to evaluate BH4 responsiveness (Table 1 and Table S1). BH4 loading tests were conducted from 8 to 48 h in most studies (but for 1 week to 4 months in 4 studies [16,29,48,49]), and the dose of BH4 prescribed ranged from 5 to 24 mg/kg/day. BH4 therapy was started at a mean age between 5 months and 18 years. Mean duration of follow-up ranged from 3 months to 5.7 years, with some patients on BH4 treatment for up to 8.8 years [17] (Table 1).

### 3.3. Systematic Review of Key Findings and Meta-Analyses

Table 2 and Table S2 summarize the main outcomes of the studies included in the systematic review, i.e., the changes in Phe and protein intakes with long-term (≥3 months) BH4 treatment. Meta-analyses of the data were performed, and the overall effect estimate is presented (SMD with confidence intervals (CI)) and illustrated in forest plots. 

#### 3.3.1. Change in Phe Intake with BH4 Treatment

**Figure 2:**
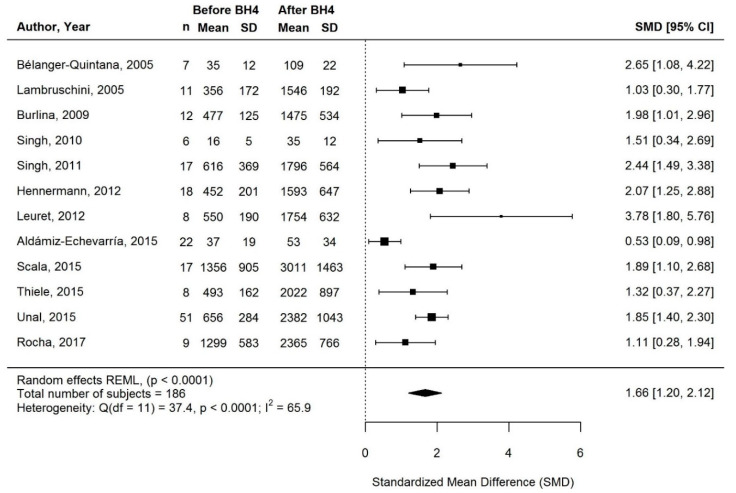
Standardized change in phenylalanine intake of long-term responders on BH4 treatment. Means and SDs before/after BH4 are milligram phenylalanine per kilogram bodyweight per day for Belanger-Quintana (2005), Singh (2010), and Aldámiz-Echevarría (2015), and milligram per day for all other studies. Abbreviations: BH4, tetrahydrobiopterin; CI: confidence interval; n: sample size; SD: standard deviation; SMD, standardized mean difference.

Long-term changes in Phe intake were evaluated in 13/18 studies. Phe intakes were self-reported in most studies (self-reported data, *n* = 10; both self-reported and prescribed data, *n* = 2; not specified, *n* = 1; Table 2). Meta-analysis of 12/13 studies showed that Phe intake increased significantly with BH4 treatment (SMD [95% CI] = 1.66 [1.20, 2.12]; *p* < 0.0001; I^2^ = 65.9%; *n* = 186 subjects; Figure 2). The effect was consistent across studies (Figure 2 and Table 2). Although only a small increase in Phe intake (≈1.5-fold) was reported in 2/12 studies [37,54], improvement was seen in 90% of long-term responders, and Phe intake increased >2-fold (range: 2.2 to 4.3-fold) in the other 10/12 studies (increase observed in 100% of long-term responders). The study that could not be included in the meta-analysis (no means and SDs) [36] showed only small increases in median Phe intake, and no change in Phe intake was observed for 22% and 40% of long-term responders after 2 and 5 y of BH4 treatment, respectively (Table 2).

#### 3.3.2. Change in Natural Protein Intake with BH4 Treatment

**Figure 3:**
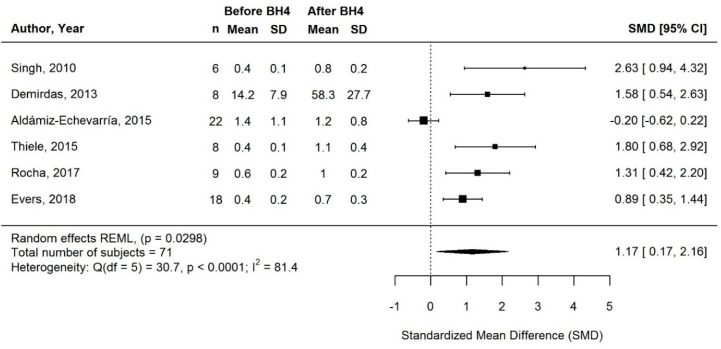
Standardized change in natural protein intake of long-term responders on BH4 treatment. Means and SDs before/after BH4 are gram natural protein per day for Demirdas (2013), and gram per kilogram bodyweight per day for all other studies. Abbreviations: BH4, tetrahydrobiopterin; CI: confidence interval; n: sample size; SD: standard deviation; SMD, standardized mean difference.

Only 7/18 studies assessed long-term changes in natural protein intake (self-reported data, *n* = 6; prescribed data, *n* = 1; Table 2). Meta-analysis of 6/7 studies demonstrated a significant increase with BH4 treatment (SMD [95% CI] = 1.17 [0.17, 2.16]; *p* = 0.0298; I^2^ = 81.4%; *n* = 71 subjects; Figure 3). The effect was consistent across 5/6 studies, although heterogeneity was high and effect sizes varied widely (range: 51 to 157% when considering the increase from baseline in g natural protein/kg/day and 79 to 311% in g/day). The remaining two studies (one not included in the meta-analysis [36]), from the same Spanish metabolic centers, showed little to no change in natural protein intake after 1 to 5 y of BH4 treatment [36,37] (Table 2).

#### 3.3.3. Change in Protein Equivalent Intake from Protein Substitute with BH4 Treatment

**Figure 4:**
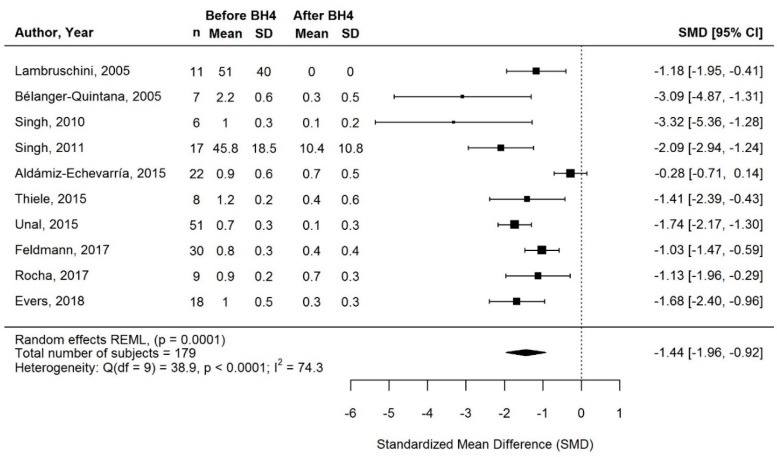
Standardized change in protein equivalent intake from protein substitute of long-term responders on BH4 treatment. Means and SDs before/after BH4 are gram protein equivalent per day for Lambruschini (2005) and Singh (2011), and gram per kilogram bodyweight per day for all other studies. Abbreviations: BH4, tetrahydrobiopterin; CI: confidence interval; n: sample size; SD: standard deviation; SMD, standardized mean difference.

Protein equivalent intake from protein substitute was self-reported in most studies (self-reported data, *n* = 13; prescribed data, *n* = 1; both, *n* = 1; not specified, *n* = 3; Table 2). Meta-analysis of 10/18 studies showed a significant, consistent reduction in protein equivalent intake from protein substitute (SMD [95% CI] = −1.44 [−1.96, −0.92]; *p* = 0.0001; I^2^ = 74.3%; *n* = 179 subjects; Figure 4). The result did not change when Belanger-Quintana et al. [38] and Singh et al. [49] were excluded in a sensitivity analysis (data not shown). This result was also broadly consistent with the findings in the remaining studies not included in the meta-analysis (Table 2). Overall, long-term BH4 treatment led to a mean decrease in protein equivalent intake from protein substitute (both when expressed as mg/day and mg/kg/day) of at least 80% compared with baseline in 9/18 studies, and at least 40% in 5/18 studies. However, the decrease in protein equivalent intake from protein substitute was <25% in 2/18 studies, and almost all patients continued to require a substantial amount of protein substitutes in both studies, despite BH4 treatment [37,54] (Table 2). For 2/18 studies, the reduction in protein equivalent intake from protein substitute could not be estimated [17,47] (Table 2).

Thereby, approximately half of all long-term responders (149/306) continued to require protein substitutes with BH4 treatment, and half (157/306) stopped protein substitute usage (Table 2). For 63/149, the dose of protein substitute was reduced in 67% (*n* = 42) but remained unchanged in 33% (*n* = 21) on long-term BH4 treatment (Table 2). In the other 86/149 patients still requiring protein substitutes, it was unreported if the amount could be decreased or remained unchanged [16,17,36,37] (Table 2).

**Table 2:** Overview of study results: changes in phenylalanine and protein intakes (total protein, natural protein, and protein equivalent from protein substitute) of long-term responders on tetrahydrobiopterin (BH4) treatment ^1^.

Reference	Duration on BH4 (Mean or Range; Years)	Change in Phe Intake		Relative Change in Natural Protein Intake from Baseline ^2^	Change in Protein Equivalent Intake from Protein Substitute		Relative Change in Total Protein Intake from Baseline ^2^
		Relative Change from Baseline ^2^	No. of Responders with Increased Intake (%)		Relative Change from Baseline ^2^	No. of Responders with Change in Dose (%) ^3^	
Bélanger-Quintana 2005 [38]	**0.9** (range: 0.4–1.5)	**3.5-fold 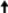 ** *(mean; mg/kg/day)* **2.7-fold 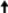 ** *(median; mg/kg/day)*	**7/7 (100)**	n/a	**90% 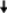 ** *(mean; g/kg/day)* **100% 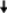 ** *(median; g/kg/day)*	Decreased: **2/7 (29)** Stopped: **5/7 (71)** No change: -	n/a
Lambruschini 2005 [45]	**1.0**	**4.3-fold 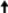 *** *(mean SR intake; mg/day)*	**11/11 (100)**	n/a	**100% 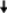 ** *(mean and median; g/day)*	Decreased: - Stopped: **11/11 (100)** No change: -	n/a
Burlina 2009 [40]	**3.5** (range: 0.5–7.0)	**3.2-fold 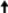 ** *(mean SR intake; mg/day)*	**12/12 (100)**	n/a	**100% 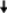 ** *(mean and median; g/day)*	Decreased: - Stopped: **12/12 (100)** No change: -	n/a
Singh 2010 [49]	**2.0**	*3mo FU:* **2.2-fold 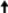 *** *1y FU:* **2.3-fold 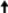 *** *2y FU:* **2.2-fold 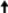 *** *(mean SR intake; mg/kg/day)* *3mo FU:* **3.4-fold 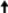 *** *1y FU:* **3.4-fold 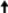 *** *2y FU:* **3.1-fold 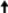 *** *(mean prescription; mg/kg/day)*	**6/6 (100)**	*3mo FU:* **114% 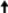 *** *1y FU:* **119% 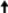 *** *2y FU:* **125% 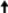 *** *(mean SR intake; g/kg/day)*	*3mo FU:* **77% 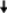 *** *1y FU:* **70% 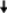 *** *2y FU:* **84% 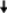 *** *(mean SR intake; g/kg/day)*	*3mo FU:* Decreased: **3/6 (50)** Stopped: **3/6 (50)** No change: - *2y FU:* Decreased: **4/6 (67)** Stopped: **2/6 (33)** No change: -	*3mo FU:* **25%** 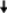 **^ns^** *1y FU:* **18%** 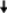 **^ns^** *2y FU:* **27%** 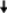 **^ns^** *(mean SR intake; g/kg/day)*
Vilaseca 2010 [51]	**5.7** (range: 5.3–6.0)	n/a	n/a	n/a	**100% 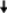 ** *(mean and median; g/day)*	Decreased: - Stopped: **10/10 (100)** No change: -	n/a
Singh 2011 [48], Douglas 2013a [42], Douglas 2013b [43], Brantley 2018 [39]	**1.0**	*4mo FU*: **2.7-fold** 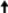 * **^†^** *(mean prescription; mg/day)* *1y FU:***2.9-fold** 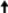 *(mean prescription; mg/day)* *1y FU:***1.5-fold** 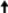 **^ns^** *(median SR intake; mg/day)*	*4mo FU*: **18/18 (100)** *1y FU:***17/17 (100)**	n/a	*4mo FU*: **83% 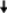 *** (mean prescription; g/day) *1y FU:***77% 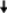 ** *(mean prescription; g/day)* **75 to 100% 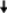 (*n* = 6/17)** **50 to 75% 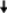 (*n* = 8/17)** **20 to 25% 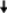 (*n* = 1/17)** **<20% 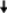 (*n* = 2/17)** *(prescription; g/day)*	*4mo FU:* Decreased: **7/18 (39)** Stopped: **9/18 (50)** No change: **2/18 (11)** *1y FU:* Decreased: **10/17 (59)** Stopped: **5/17 (29)** No change: **2/17 (12)**	n/a
Hennermann 2012 [17]	**4.0** (range: 0.7–8.8)	**3.8-fold 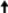 ** *(mean; mg/day)* **3.1-fold 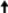 ** *(median; mg/day)*	**18/18 (100)**	n/a	n/a	Decreased/ No change: **10/18 (56)** Stopped: **8/18 (44)**	n/a
Leuret 2012 [46]	**1.9 ^§^** (range: 0.6–6.7)	**3.2-fold 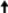 *** *(mean SR intake; mg/day)*	**8/8 (100)**	n/a	n/a	Decreased: - Stopped: **7/8 (87)** No change: **1/8 (13)**	n/a
Aldámiz-Echevarría 2013 [36]	**2.0** (cohort 1) ^#^ **5.0** (cohort 2) ^#^	*2y FU:* **1.4-fold 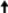 ** *(median SR intake; mg/kg/day)* *5y FU:* **1.2-fold 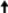 ** *(median SR intake; mg/kg/day)*	*2y FU:* **28/36 (78)** *5y FU:* **6/10 (60)**	*2y FU:* **14% 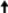 ** *(median SR intake; g/kg/day)* *5y FU:* **13% 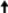 ** *(median SR intake; g/kg/day)*	*2y FU:* **44% 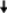 ** *(median SR intake; g/kg/day)* *5y FU:* **57% 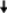 ** *(median SR intake; g/kg/day)*	*2y FU:* Decreased/ No change: **25/36 (69)** Stopped: **11/36 (31)** *5y FU:* Decreased/ No change: **8/10 (80)** Stopped: **2/10 (20)**	*2y FU:* **17% 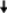 ** *(median SR intake; g/kg/day)* *5y FU:* **29% 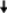 ** *(median SR intake; g/kg/day)*
Demirdas 2013 [41]	range: **1.4–2.0**	n/a	**8/8 (100)**	**311% 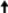 *** *(mean SR intake; g/day)*	**100% 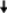 (*n* = 3/8)** **>60% 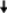 (*n* = 3/8)** **<20% 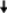 (*n* = 2/8)** *(SR intake; g/day)*	Decreased: **5/8 (63)** Stopped: **3/8 (37)** No change: -	n/a
Aldámiz-Echevarría 2015 [37]	**1.0**	**1.4-fold 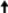 *** *(mean SR intake; mg/kg/day)*	**20/22 (90)**	**14% 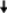 ^ns^** *(mean SR intake; g/kg/day)*	**22% 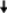 ^ns^** *(mean SR intake; g/kg/day)*	Decreased/ No change: **20/22 (91)** Stopped: **2/22 (9)**	**14% 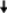 ^ns^** *(mean SR intake; g/kg/day)*
Scala 2015 [47]	**5.7** (range: 1.0–7.0)	**2.5-fold 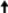 *** *(mean SR intake; mg/day)* **2.7-fold 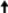 *** *(median SR intake; mg/day)*	**17/17 (100)**	n/a	n/a	Decreased: **2/17 (12)** Stopped: **9/17 (53)** No change: **6/17 (35)**	n/a
Thiele 2015 [29]	**2.0**	*3mo FU:* **4.5-fold 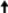 *** *2y FU:* **4.1-fold 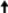 *** *(mean SR intake; mg/day)*	**8/8 (100)**	*3mo FU:* **307% 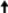 *** *(g/day)* **244% 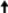 *** *(g/kg/day)* *(median SR intake)* *2y FU:* **244% 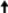 *** *(g/day)* **157% 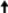 *** *(g/kg/day)* *(median SR intake)*	*3mo FU:* **100% 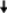 *** *(g/day)* **100% 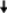 *** *(g/kg/day)* *(median SR intake)* *2y FU:* **84% 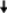 *** *(g/day)* **88% 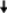 *** *(g/kg/day)* *(median SR intake)*	Decreased: - Stopped: **4/8 (50)** No change: **4/8 (50)**	*3mo FU:* **12% 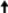 ^ns^** (*g/day)* **4% 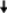 ^ns^** *(g/kg/day)* *(median SR intake)* *2y FU:* **27% 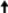 ^ns^** (*g/day)* **2% 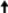 ^ns^** *(g/kg/day)* *(median SR intake)*
Ünal 2015 [50] Gökmen Özel 2014 [52]	**2.5** (range: 0.5–4.0)	**3.8-fold 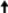 ** *(mg/day)* **2.9-fold 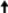 ** *(mg/kg/day)* *(mean SR intake)* **3.7-fold 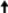 ** *(mg/day)* **2.8-fold 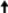 ** *(mg/kg/day)* *(median SR intake)*	**51/51 (100)**	n/a	**87% 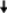 ** *(g/day)* **89% 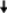 ** *(g/kg/day)* *(mean SR intake)* **100% 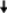 ** *(g/day)* **100% 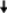 ** *(g/kg/day)* *(median SR intake)*	Decreased: **5/51 (10)** Stopped: **43/51 (84)** No change: **3/51 (6)**	**79% 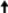 ** *(g/day)* **35% 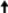 ** *(g/kg/day)* *(mean SR intake)* **78% 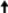 ** *(g/day)* **33% 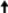 ** *(g/kg/day)* *(median SR intake)*
Feldmann 2017 [16]	**0.5**	n/a	n/a	n/a	**49% 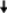 ** *(mean; g/kg/day)*	Decreased/ No change: **23/30 (77)** Stopped: **7/30 (23)**	**92% 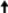 ** *(mean; g/day)*
Rocha 2017 [54]	**1.0** (range: 0.3–1.4)	**1.8-fold 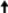 ** *(mg/day)* **1.5-fold 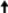 ** *(mg/kg/day)* *(median SR intake)*	**8/9 (89)**	**79% 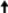 ** *(g/day)* **51% 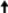 ** *(g/kg/day)* *(median SR intake)*	**16% 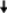 ** *(g/day)* **23% 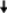 ** *(g/kg/day)* *(median SR intake)*	Decreased: **4/9 (44)** Stopped: - No change: **5/9 (56)**	**19% 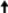 ** *(g/day)* **8% 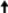 ** *(g/kg/day)* *(median SR intake)*
Evers 2018 [44]	**5.0** (range: 4.5–5.5)	n/a	n/a	**59% 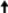 ** *(mean prescription; g/kg/day)* **100% 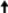 ** *(median prescription; g/kg/day)*	**69% 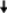 ** *(mean prescription; g/kg/day)* **61% 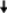 ** *(median prescription; g/kg/day)*	Decreased: **10/18 (56)** Stopped: **8/18 (44)** No change: -	**33% 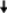 ** *(mean prescription; g/kg/day)*
Paras 2018 [53]	**≥0.3** (range: ≥0.3–≥3.5)	n/a	**8/8 (100)**	n/a	**100% 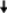 ** *(mean and median; g/day)*	Decreased: - Stopped: **8/8 (100)** No change: -	n/a

Abbreviations: FU: follow-up; No: number; ns: not statistically significant; Phe: phenylalanine; SR: self-reported; y: year; mo: month; n/a: not available. 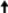: increase; 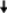: decrease. ^1^ Only long-term responders (follow-up ≥3 months) who were on a Phe-restricted diet and protein substitute before BH4 were included in the analyses. Long-term responsiveness as reported by the original authors, except for Rocha 2017 where 1 patient was considered long-term non-responder after discussing with the authors (lack of changes in Phe tolerance and natural protein intake, while Phe levels only decreased by 10%). ^2^ Superscripts indicate that a statistical analysis was performed by the original authors. *: statistically significant change; ^ns^: change not statistically significant. Otherwise, no statistical analysis was performed with the exception of Ünal 2015, Rocha 2017, and Evers 2018, who performed statistical analyses with their original samples. However, statistical significance is not reported here because some patients included in the original analyses did not meet our inclusion criteria (i.e., long-term responders followed up ≥3 months who were on a Phe-restricted diet and protein substitute before BH4). ^3^ Change as reported by the original authors. If individual data were available (i.e., reported or provided upon request), change in protein substitute intake was considered a “decrease” only if the reduction was ≥25% compared with baseline, as this was deemed clinically meaningful. Reductions <25% of baseline were counted as “no change”. ^†^ Singh 2011: Change in Phe tolerance at 4mo FU included 1 patient never taking any protein substitute but who could not be removed from this analysis, and thus *n* = 19 instead of 18. One other patient was lost to follow-up between 4mo and 1y FU. ^§^ Leuret 2012: Median duration of BH4 treatment, not mean. Only 8/15 patients were on a Phe-restricted diet before BH4 and were therefore included in our analyses; however, duration of BH4 treatment was only available for the total sample of 15 patients. ^#^ Aldámiz-Echevarría 2013: Unclear if patients with a 5y follow-up were also described in the group of patients with a 2y follow-up. It was assumed that the 2 cohorts comprised different patients.

#### 3.3.4. Change in Total Protein Intake after BH4 Treatment

**Figure 5:**
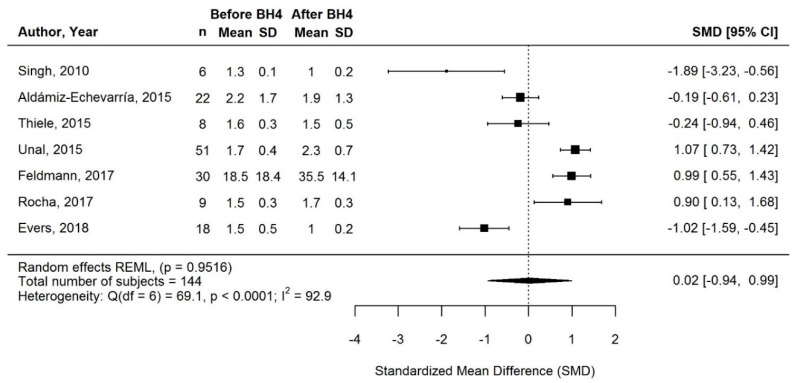
Standardized change in total protein intake of long-term responders on BH4 treatment. Means and SDs before/after BH4 are gram total protein per day for Feldmann (2017), and gram per kilogram per day for all other studies. Abbreviations: BH4, tetrahydrobiopterin; CI: confidence interval; n: sample size; SD: standard deviation; SMD, standardized mean difference.

Only 8/18 studies evaluated long-term changes in total protein intake (Table 2), and meta-analysis of 7/8 studies showed no significant change with BH4 treatment (SMD [95% CI] = 0.02 [−0.94, 0.99]; *p* = 0.9516; I^2^ = 92.9%; *n* = 144 subjects; Figure 5). However, there was a considerable amount of heterogeneity within the data. Although results across studies were inconsistent, the mean/median total protein intakes (per kg of body weight) met dietary reference values for protein intake throughout the evaluation periods [55].

#### 3.3.5. Supplementary Sensitivity Meta-Analyses

Some authors reported dietary/nutritional outcomes in gram per day (mg/day for Phe intake), whereas others expressed their results per kilogram bodyweight (g/kg/day or mg/kg/day), and thus SMDs were used in the main meta-analyses in order to compare data in different units. However, for each dietary outcome, two sets of meta-analyses were also performed by pooling only studies expressing data in the same unit (Figures S1–S8). Despite the generally high heterogeneity within the data, results were similar irrespective of the units used and in line with the main meta-analyses reported above. One exception was total daily protein intake, where, although no significant change was observed per kilogram bodyweight, total protein intake significantly increased by 16.71 g/day with BH4 treatment (95% CI = [6.91, 26.50]; *p* = 0.0123; I^2^ = 73.9%; 4 studies; *n* = 98 subjects; Figure S8). Finally, because of the particularly high heterogeneity in the meta-analyses of the changes in milligram Phe intake per kilogram bodyweight per day (Figure S1; I^2^ = 96.4) and gram protein equivalent intake from protein substitute per kilogram bodyweight per day (Figure S5; I^2^ = 97.5), sensitivity analyses excluding Belanger-Quintana et al. [38] were performed; however, results remained similar (data not shown).

### 3.4. Systematic Review of Findings Related to Secondary Outcomes

#### 3.4.1. Change in Micronutrient Intakes and Serum Concentrations with BH4

Only 8/18 studies investigated the change in micronutrient intakes [17,29,39,45,49] and/or markers of nutritional status [17,39,44,45,47,49,54] with long-term BH4 treatment (data not shown). Thiele et al. reported significant decreases in vitamin (OH)D_3_, vitamin B_12_, folic acid, iron, and calcium intakes, and in one patient, protein substitute had to be re-introduced because of severe atopic skin lesions, lowering of serum zinc concentration below normal range, and decreased protein intake below 80% of the recommended amount [29]. Similar changes in intakes of these micronutrients were reported by Brantley et al., along with significant decreases in serum iron, folate, and vitamin B_12_ concentrations compared to baseline [39]. Diet was not fully liberalized in all patients, but protein substitute intake was reduced by at least 50% in both studies. Lower intakes of calcium, iron, and vitamin B_12_ were also observed by Hennermann et al. [17], but only in patients who could liberalize their diet without protein substitute, and serum levels remained within the normal range. In contrast, other authors found no significant change in dietary intakes or serum concentrations of several micronutrients [44,45,47,49], except for a decrease in zinc concentrations in 5 patients in one study [54].

#### 3.4.2. Change in Growth with BH4

Of the 18 studies, 9 investigated changes in weight and height z-scores during long-term BH4 treatment (data not shown). In general, weight- and height-for-age z-scores remained within the normal range [17,29,38,44,45]. Improvement in linear growth was observed in two studies after diet liberalization with BH4 treatment, which may be attributable to a marked increase in Phe/natural protein intake [49,52]. In two other studies, weight and height z-scores were below average at baseline (z-scores < 0) and did not improve after 1 to 5 years of BH4 treatment. In both studies, the increase in Phe intake was limited (<1.5-fold), while protein equivalent intake from protein substitute intake was reduced by 22–57%, resulting in slight decrease in total protein intake [36,37].

#### 3.4.3. Change in Metabolic Control with BH4

Of the 18 studies, 15 evaluated metabolic control after BH4 treatment (data not shown). Overall, blood Phe concentrations did not change compared to baseline in 8/18 [17,36,37,38,44,45,49,50], significantly increased in 2/18 [29,47], and decreased in the remaining 5/18 studies [40,46,48,53,54]. Mean/median blood Phe levels remained in age-specific therapeutic ranges in most subjects. In one study [40], long-term BH4 treatment was only started in initial responders who were non-adherent with the low-Phe diet and had a baseline blood Phe level higher than the recommended range. At last follow-up (range: 6 months to 7 years), blood Phe levels had lowered into the therapeutic range in all subjects, and their diet was liberalized.

### 3.5. Quality Appraisal and Risk of Bias Assessment

Overall, the quality was rated as “fair” for most studies (13/18) (Table 3). The main concerns were small sample sizes and likely selection bias, making it unclear if the study samples were representative of PKU patients who would benefit from long-term BH4 treatment. A statistical analysis for pre–post treatment comparisons was also lacking in most cases. Three studies with low risks of bias were rated as “good quality” [44,48,50]. The remaining two studies were judged “poor” due to unreliability or inadequacy of outcome measurements, serious selection bias, small sample size, and lack of information on the intervention (i.e., BH4 treatment) [41,53].

## 4. Discussion

This is the first time that changes in protein equivalent intake from protein substitute with BH4 treatment have been assessed systematically, although other systematic reviews or meta-analyses have investigated the effects of BH4 treatment on blood Phe control and dietary Phe tolerance [56,57,58]. We have demonstrated that PKU patients with long-term BH4 responsiveness had a significant increase in dietary Phe and natural protein intake when on BH4 treatment. This enabled the majority of responsive patients to reduce the dose of protein substitute, and 51% (157/306) were able to stop protein substitute. However, almost half (149/306) of long-term responders continued to require some protein substitute, even though Phe and natural protein tolerance substantially improved. In this group, the protein substitute dose could be reduced in 28% (42/149) but remained unchanged in 14% of patients (21/149). In 58% (86/149) of patients on BH4 with protein substitute, the authors did not report if the dose was adjusted. Overall, the extent of reduction of protein equivalent intake from protein substitute, the time needed for change, as well as approaches to adjusting the PKU diet varied widely between studies. These findings highlight the need for guidance on when and how to decrease or stop protein substitute intake with BH4 treatment.

Pooled analysis of 10 studies showed that protein equivalent intake from protein substitute significantly decreased after a median BH4 treatment of one year (range: 0.5–5 years). Where half or more of the responsive patients were able to reduce or stop the use of protein substitutes, dietary Phe tolerance (as either expressed in mg/kg/day or mg/day) had increased by 2.5- to 4.3-fold [29,38,40,45,46,47,48,49,50]. In contrast, three studies reported a Phe tolerance increase <1.5-fold [36,37,54], and two of them failed to show a meaningful reduction (i.e., ≥25% from baseline) in median [54] or mean [37] protein equivalent intake from protein substitute after 1 year of BH4 treatment. Aldámiz et al. [37] attributed these findings to the inability of the BH4 loading test “cut off” of 30% decrease in blood Phe concentrations to identify true (i.e., long-term) responders correctly. When a 50% decrease in blood Phe as cut-off was used in a new loading test protocol [59], all responders were able to consume normal diets without protein substitute in the long term [37]. Most studies included in this systematic review used ≥30% decrease in blood Phe levels as a criterion to define BH4 responsiveness and showed successful long-term outcomes. However, BH4 therapy was discontinued in some patients (*n* = 27) mainly due to unsatisfactory blood Phe control when additional Phe/natural protein was added longer term [16,17,45,47,48,50,60].

Meeting nutritional requirements while maintaining blood Phe concentrations within therapeutic range is a central consideration when prescribing pharmaceutical therapies for PKU. Daily protein and micronutrient requirements increase throughout childhood and in women during pregnancy and lactation. With BH4 treatment, it is important to use a stepwise approach to increasing natural protein whilst in parallel reducing protein equivalent intake from protein substitute by similar amounts. Attention should be paid to the quantity as well as quality of natural protein. It is critical to ensure a good mix of animal and plant protein so that natural foods can supply all the nutrients in the amounts that meet requirements. Ongoing evaluation about the need for protein substitute supplementation as well as education about appropriate food choices is essential. We identified only a few studies [17,45,48] that have described in detail how natural protein is increased with BH4 therapy (see Table S1). Of these, the protocol by Singh et al. (2011) was the most thorough [48]. All responsive patients were instructed to add 20g of non-fat dry milk powder (≈350 mg Phe or 6.8 g protein) to their diet each week until new Phe tolerance was established [48], although this may be considered a rapid increase in natural protein intake by some. In practice, it may take several months to determine the final Phe tolerance and establish the ongoing need for a source of protein equivalent from protein substitute. Paras et al. reported a range of 3 months to 3.5 years until full diet liberalization occurred [53]. Caution is necessary in the case of illness episodes, injury, or trauma, as these may all adversely affect metabolic control, and it is established that BH4 is less effective in illness [38]. Protein substitutes offer a protective role by counteracting protein catabolism. It may be considered that, in young children, a small dose of protein substitute should be maintained as it is difficult to re-establish intake specifically for illness episodes or to meet the increased age-appropriate protein requirements during growth phase [61,62]. For others, it will be necessary to evaluate the need for protein substitute re-introduction or an increase in dose might be required. Some studies have described patients who could initially stop using protein substitute, but for whom it had to be re-introduced [29,48].

Most protein substitutes provide a major supply of vitamins and minerals, and one of the concerns associated with long-term BH4 treatment is the nutritional adequacy of a relaxed diet when protein substitute is stopped or reduced [29]. We found inconsistent results about the impact on micronutrient status. Overall, the reduction in usage of protein substitutes or change in dietary habits with BH4 led to a decreased intake of several essential micronutrients in some [17,29,39,54] but not all studies [44,45,47,49]. Nutritional inadequacies were generally observed when diet was not fully liberalized, particularly when the dose of protein substitute was reduced by at least half of the baseline prescription [29,39], but it was also reported in a subgroup of patients who could relax their diet and stop protein substitute intake [17]. Another concern has been the establishment of healthy eating habits in BH4-treated patients who were well established in their dietary patterns before initiation of BH4 therapy. One of the two studies that investigated change in eating habits after diet relaxation demonstrated poorer eating habits in patients treated with BH4, despite training and education [29]. Although there was some recovery (e.g., re-increase of fruit intake) after 2 years of treatment, consumption of fish and dairy products remained markedly lower than healthy peers and was replaced by a higher intake of potatoes and pasta [29]. Similar findings were also reported by Hennermann et al. [17] who observed that normal bread, normal pasta, eggs, sausages, and meat were well accepted when dietary treatment was relaxed, while milk and dairy products were poorly accepted, and fish was completely refused by all patients. Growth impairment was found only in 2/9 studies [36,37]. This was evident at baseline and it did not improve with BH4 therapy, possibly due to the limited increase in dietary Phe tolerance coupled with a slight decrease in protein equivalent from protein substitute and thus total protein intake. Overall, our results indicate that long-term BH4 therapy does not seem to have a negative impact on total protein intake, and hence on growth. Nonetheless, there is still a risk of inadequate protein quality and of micronutrient deficiencies, which may be attributable to an embedded high-carbohydrate, low-protein disordered eating pattern that may take many months and years of education and counselling to improve. Further investigations in larger prospective studies including patients from different age groups and with all forms of PKU are needed to confirm the effects of BH4 treatment on dietary adequacy and growth.

### Strengths and Limitations

The main strength of this systematic review and meta-analysis is that we only included patients who demonstrated long-term BH4 responsiveness. Some patients who appeared BH4-responsive immediately following a loading test in the long-term were unable to increase their Phe tolerance/natural protein intake without a detrimental impact on metabolic control [16,17,45,47,48,50,60]. In this patient category, protein substitute prescription usually remained unchanged, and if dose was decreased, a later increase was necessary. We decided to exclude these patients (i.e., long-term non-responders) in order to evaluate the impact of BH4 supplementation on change in protein equivalent intake from protein substitute in patients for whom the drug was “justly” efficacious. Furthermore, we believe that the duration of follow-up strengthens the reliability of these findings. We elected to include only studies where patients had been on BH4 for at least 3 months. In fact, the majority (55%) of studies included had a mean BH4 treatment duration of ≥2 years, with some patients on cofactor therapy for almost 9 years [17].

Our work also had several limitations. Many articles were excluded during the screening process due to inadequate information about protein substitute intake (47/62). It is crucial in any study investigating new treatments for PKU to measure and report any changes in protein intake (including both natural and protein equivalent from protein substitute). Furthermore, one of the inclusion criteria was that prior to BH4 treatment, a Phe-restricted diet supplemented with protein substitute was necessary, which led to the exclusion of a limited number of patients on a normal diet at baseline from the analyses. The meta-analyses showed a medium-to-high level of heterogeneity between study results for the main outcomes of interest. This may be explained by the wide differences in age and phenotypes of patients, as well as the variation in the definition of BH4 responsiveness, duration of follow-up, target blood Phe levels, or the protocols followed by centers for dietary changes with BH4. Authors usually described self-reported intakes rather than prescribed amounts of protein. Non-adherence to the prescribed amount of protein substitute is common in PKU, and hence the change in self-reported intakes may not reflect the true effect of BH4. Finally, the quality of most included studies was rated as fair only for several reasons, e.g., small sample size, lack of power analysis, or absence of statistical comparison, even though some of these limitations are due to the rarity of the disorder.

## 5. Recommendations

This work, as well as our clinical experience, call for several recommendations, which will help guide healthcare professionals when adjusting dietary prescriptions of patients with PKU on BH4 treatment. Some of these recommendations will also be valid for other new therapies such as pegvaliase.

### 5.1. BH4 Treatment Trial and Adjusting Phe Intake

- BH4 responsiveness requires careful assessment—the aim is to maintain blood Phe within target therapeutic range while maintaining normal growth but also (1) establish an increase in Phe tolerance, (2) reduce protein equivalent intake from protein substitute in alignment with any increase in natural protein intake, and (3) establish the maintenance dose of BH4.
- Once BH4 is administered, if three consecutive blood Phe levels are maintained within target therapeutic range, then Phe intake should be increased by at least 20%, and then this process should be repeated until natural protein tolerance is established. If the mean blood Phe level exceeds target therapeutic range, then the Phe intake should be reduced by approximately 10 to 30%, depending on the degree of elevation of the blood Phe levels (adapted from Muntau et al. [63]).
- With BH4 treatment, it is expected that the final Phe tolerance should be increased by ≥100% of baseline, provided natural protein intake is below safe levels of protein intake. If natural protein intake already exceeds safe levels of protein intake at baseline, an improvement in blood Phe control may be an appropriate alternative goal. Maintenance of blood Phe levels within target therapeutic range and an increase in Phe tolerance should be observed for at least 3 months to ascertain BH4 responsiveness.

### 5.2. Quality of Natural Protein Intake

- Natural protein intake should be sourced from different proteins, e.g., dairy and eggs, cereals, lentils, and protein-rich vegetables if tolerated. Food choices should be made according to national and international recommendations. Natural protein sources should provide micronutrients to minimize the need for extra micronutrient supplements. Continuous patient education and support about the need for a healthy diet with appropriate food choices will be necessary with BH4 treatment.

### 5.3. Adapting Protein Substitute Dose

- Protein equivalent from substitute intake should be reduced in parallel with any increase in natural protein intake. The more natural protein that is tolerated, the lower the requirement should be for protein substitute. For every increase in natural protein, the protein equivalent from protein substitute should be reduced accordingly.
- It is possible that the natural protein intake meets or exceeds safe levels of protein intake so that a protein substitute is not needed to meet protein requirements. However, some protein substitute might be necessary for micronutrient requirements to be met. Micronutrient supply should be monitored carefully, especially if patients cannot be allowed an unlimited Phe intake. Moreover, it may be better for patients to remain familiar with and accepting of the taste of protein substitute in case it needs to be reintroduced in illness, pre-conception, pregnancy, or lactation, or if BH4 therapy is discontinued. It is also good practice to give a small dose of protein substitute each day to infants who may appear fully responsive to BH4 and without immediate need for a protein restriction. It is possible protein restriction may be necessary at a later age when daily protein requirements increase.

### 5.4. Monitoring

- Once patients are established on BH4 therapy and the diet is stabilized, clinic visits and blood monitoring should occur at the same frequency as for other patients with PKU who are not on BH4 treatment. If there are any concerns about adherence with BH4 or diet, more frequent monitoring may be required.
- Continue to assess that at least 75% of blood Phe levels remain within target therapeutic range and that more than 100% of original prescription of Phe intake is maintained (unless patients are already meeting safe levels of protein intake). If more than 25% of blood Phe levels are outside target therapeutic range, consider adjusting BH4 dosage or reduce Phe intake. BH4 treatment continuation should be evaluated.
- Evaluate if protein substitute should be re-introduced, or prescription increased, in any event of increased protein requirements (rapid growth, illness, injury/trauma, pregnancy, lactation).
- Patient’s nutritional status including height/length, weight, and body mass index (BMI) should be conducted at least 6-monthly. It is important that patients are encouraged to maintain a healthy BMI.
- Assessment of patient’s nutritional biochemical markers such as plasma amino acids, homocysteine/or methyl malonic acid, hemoglobin, mean corpuscular volume, ferritin, zinc, calcium, selenium, vitamin D, vitamin B12, and folic acid should be completed annually for patients on BH4 therapy.
- Monitor nutritional intake adequacy by 3-day dietary assessments regularly, at least every 3 months in the first year of BH4 therapy. Vitamin and mineral supplements may be required if dietary assessment or patient’s nutritional biomarkers indicate they are necessary. Patients may be more vulnerable to nutritional deficiency if they have stopped or reduced protein substitute intake.
- The ongoing prescription for BH4 should be reassessed and adjusted as appropriate at each clinic visit.

### 5.5. Clinical Trials of (New) Treatments

- Any future studies investigating treatment strategies for PKU should evaluate long-term (at least 6 months) changes in nutrient intake, in particular natural protein, the need for protein substitute, and micronutrient supplementation. Data about prescribed as well as self-reported protein/Phe intakes should be collected and reported (both gram (or milligram) per day and gram (or milligram) per kilogram bodyweight per day). In published studies, individual data should be provided rather than only summary statistics such as means or medians.

## 6. Conclusions

In BH4-responsive patients with PKU, protein equivalent intake from protein substitute significantly decreased with long-term BH4 treatment, with half of the patients able to stop protein substitute and follow a liberalized diet. However, the other half of BH4 responders still required at least some protein substitute to meet their protein requirements and to achieve good metabolic control, even though Phe tolerance substantially improved. It is important to follow a systematic protocol to increase natural protein intake while reducing the dose of protein substitutes in order to ensure protein and micronutrient requirements are met and sustained. Normal growth was maintained with BH4 treatment, but micronutrient deficiency associated with a decreased intake of protein substitute is a potential risk. Special attention is required in any situations where protein requirements are increased (e.g., rapid growth, illness, or pregnancy), and increase in prescription or re-introduction of protein substitute should be evaluated.

## Figures and Tables

**Table 1:** Main characteristics of included studies.

Reference	Country	Study Design	No. of Patients Tested/No. of Long-Term Responders ^a^		Gender of Long-Term Responders (M/F)	Duration of BH4 Loading Test	BH4 Dose (Mean or Range; mg/kg/day)	Age at Initiation of BH4 (Mean or Range; Years)	Duration of Follow-up (Mean or Range; Years)
Bélanger-Quintana 2005 [38]	Spain	Retrospective longitudinal single-center study	Total: mHPA: mPKU: mo/cPKU:	50/7 ^b^ 7/- 22/7 21/-	n/a	24 h	5–20 ^†^	7.8 (range: 0.7–18)	0.9 (range: 0.4–1.5)
Lambruschini 2005 [45]	Spain	Prospective longitudinal single-center study	Total: mHPA: mPKU: moPKU: cPKU:	73/11 ^c^ - -/9 -/2 -	4/7	24 h ^d^	5–10 ^†^	5.0 (range: 0.2–12.2)	1.0
Burlina 2009 [40]	Italy	Retrospective longitudinal single-center study	Total:	30/12 ^e^	n/a	24 h	10 ^†^	5.5 (range: 2.0–16.0)	3.5 (range: 0.5–7.0)
Singh 2010 [49]	USA	Prospective longitudinal single-center study	Total:	10/6 ^f^	6/0	1 week	20 ^‡^	8.7 (range: 5–12)	2.0
Vilaseca 2010 [51]	Spain	Cross-sectional single-center study	Total: mHPA: mPKU: moPKU: cPKU:	61/10 ^g^ - 5/3 21/7 35/-	n/a	21 h	5–15 ^†^	7.4 (range: 1.0–16.0)	5.7 (range: 5.3–6.0)
Singh 2011 [48] Douglas 2013a [42] Douglas 2013b [43] Brantley 2018 [39]	USA	Prospective longitudinal single-center study	Total:	57/17 ^h^	10/7	4 months	20 ^‡^	16.6 (range: 6.1–36.8)	1.0
Hennermann 2012 [17]	Germany	Prospective longitudinal single-center study	Total:	84/18 ^i^	n/a	24 h (*n* = 56) 8 h (*n* = 26)	8–19 ^§^	n/a	4.0 (range: 0.7–8.8)
Leuret 2012 [46]	France	Retrospective longitudinal multicenter study	Total: mHPA: mPKU: moPKU: cPKU:	-/8 ^j^ - -/8 - -	n/a	24 h	8–24 ^§^	1.1 (range: 0.4–2.9)	1.9 ^j^ (range: 0.6–6.7)
Aldámiz-Echevarría 2013 [36]	Spain	Retrospective longitudinal multicenter study	*Cohort 1: Patients with 2 y follow-up* ^k^						
			Total: mHPA: mPKU: moPKU: cPKU:	-/36 - -/7 -/24 -/5	18/18	24 h (24 h or 1 week at one hospital after 2005)	5–20 ^§^	5.0	2.0
			*Cohort 2: Patients with 5 y follow-up* ^k^						
			Total: mHPA: mPKU: moPKU: cPKU:	-/10 - -/1 -/9 -	6/4	24 h (24 h or 1 week at one hospital after 2005)	5–20 ^§^	5.2	5.0
Demirdas 2013 [41]	The Netherlands	Prospective multicenter cohort study	Total:	45/8 ^l^	n/a	48 h	n/a ^‡^	n/a	range: 1.4–2.0
Aldámiz-Echevarría 2015 [37]	Spain	Retrospective longitudinal multicenter study	Total: mHPA: mPKU: moPKU: cPKU:	-/22 - -/5 -/14 -/3	12/10	8 h or 12 h; (24 h or 1 week at one hospital after 2005)	5–20 ^§^	1.4 (neonatal in *n* = 4)	1.0
Scala 2015 [47]	Italy	Prospective longitudinal multicenter study	Total: mHPA: mPKU: moPKU: cPKU:	43/17 ^m^ -/3 -/8 -/4 -/2	11/6	48 h	10 ^§^	15.1 (range: 7.0–22.0)	5.7 (range: 1.0–7.0)
Thiele 2015 [29]	Germany	Retrospective longitudinal single-center study	Total: mHPA: mPKU: moPKU: cPKU:	-/8 -/3 -/3 -/1 -/1	5/3	6 weeks	10–19 ^‡^	8.8 (range: 5.0–15.0)	2.0
Ünal 2015 [50] Gökmen Özel 2014 [52]	Turkey	Cross-sectional single-center study	Total: mHPA: mPKU: moPKU: cPKU:	-/51 ^n^ -/18 -/23 -/6 -/3	27/24	48 h	20 ^‡^	5.4 (range: 0.5–14.0)	2.5 (range: 0.5–4.0)
Feldmann 2017 [16]	Germany	Prospective longitudinal single-center study	Total:	112/30 ^o^	n/a	2 weeks	20 ^‡^	n/a	0.5
Rocha 2017 [54]	Portugal	Retrospective single-center cohort study	Total: mHPA: mPKU: moPKU: cPKU:	-/9 ^p^ - - -/8 -/1	3/6	48 h	n/a ^‡^	16.6 (range: 9.0–28.0)	1.0 (range: 0.3–1.4)
Evers 2018 [44]	The Netherlands	Retrospective multicenter cohort study	Total:	-/18 ^q^	5/13	48 h	10–20 ^‡^	12.0 (range: 4.0–19.0)	5.0 (range: 4.5–5.5)
Paras 2018 [53]	USA	Retrospective longitudinal single-center study	Total:	-/8 ^r^	n/a	n/a	20 ^‡^	5.8 (range: 0.4–18.0)	≥0.3

Abbreviations: (6R-)BH4: tetrahydrobiopterin; M/F: male/female; mHPA: mild hyperphenylalaninemia; cPKU: classic phenylketonuria; mPKU: mild phenylketonuria; moPKU: moderate phenylketonuria; No: number; Phe: phenylalanine; n/a: not available. ^†^ BH4 given as 6R-BH4 (Bélanger-Quintana 2005; Lambruschini 2005; Burlina 2009; Vilaseca 2010). ^‡^ BH4 given as sapropterin dihydrochloride (Singh 2010; Singh 2011; Demirdas 2013; Thiele 2015; Ünal 2015; Feldmann 2017; Rocha 2017; Evers 2018; Paras 2018). ^§^ BH4 given as 6R-BH4 before 2009 and as sapropterin dihydrochloride after 2009 (Hennermann 2012; Leuret 2012; Aldámiz-Echevarría 2013; Aldámiz-Echevarría 2015; Scala 2015). ^a^ Only long-term responders (follow-up ≥3 months) who were on a Phe-restricted diet and protein substitute before BH4 were included in the analyses. Long-term responsiveness as judged by the original authors. ^b^ Bélanger-Quintana 2005: Long-term BH4 treatment was initiated only in 7 responders with mild PKU who were able to liberalize their diet. ^c^ Lambruschini 2005: Only 11 out of 14 responders were included in the analyses: BH4 therapy was stopped in 3 patients (1 cPKU and 2 moPKU) who were not able to increase their Phe tolerance and continued to take medical formula. ^d^ Lambruschini 2005: BH4 loading test was performed after neonatal screening before starting the Phe-restricted diet in 7 patients. A combined 24 h-long Phe/BH4 loading test was used in the remaining 66 patients. ^e^ Burlina 2009: Long-term BH4 treatment was initiated only in 12 responders who had a baseline Phe level >450 μmol/L. ^f^ Singh 2010: From a total of 7 responders, 6 male patients were included in the analyses (the female patient dropped out of the study). Age reported here is for total sample of 10 patients. ^g^ Vilaseca 2010: Only 10 out of 13 patients were included in the analyses: 3 patients (2 mPKU and 1 moPKU) were excluded since the BH4 loading test was performed just after neonatal screening before starting the Phe-restricted diet and protein substitutes. Age reported here is for the 13 patients. ^h^ Singh 2011: Thirty-two patients who experienced at least a 15% decrease in plasma Phe at 1 month were described as “preliminary responders“. Of these, 20 patients who could increase Phe tolerance by at least 300 mg/d, and decrease prescribed medical food needs by at least 25% with good metabolic control were defined as “definitive/true responders” (long-term responders). Nine patients were considered “provisional responders” (long-term non-responders: 6 males and 3 females aged between 4.6 to 17.8 years) and excluded from the analyses. Two long-term responders had dropped out by 4 months of follow-up and a third dropped out between 4 months and 1 year; hence, 18 and 17 long-term responders were included in the analyses for the 4 months and 1 year follow-ups, respectively. *n* = 17 is shown here as it was the number of responders at last follow-up. Age reported here is for the responders including a dropout and 1 patient never on protein substitute. ^i^ Hennermann 2012: Neonatal BH4 loading test was performed in 84 patients. Long-term responsiveness was described on the basis of the increase in Phe tolerance after 3 months of BH4 initiation. Only 18 out of 23 patients (11 males, 12 females) who met the criteria were included in the analyses. The other 5 patients were considered long-term non-responders. ^j^ Leuret 2012: From a total of 15 responders (7 males, 8 females), only 8 were treated by conventional diet therapy (i.e., Phe-restricted diet supplemented with protein substitutes) before initiation of BH4 and were included in the analyses. The other 7 patients who started BH4 therapy during the neonatal period were excluded. However, duration of BH4 treatment was only available for the total sample of 15 patients. ^k^ Aldámiz-Echevarría 2013: Unclear if patients with a 5 y follow-up were also described in the group of patients with a 2 y follow-up. It was assumed that the 2 cohorts comprised different patients. ^l^ Demirdas 2013: Only 8 out of 10 responders (mean age 13.8 years) with complete data on dietary intakes were included in the analyses. ^m^ Scala 2015: From a total of 19 responders, 17 were included: 2 mPKU patients who did not agree to participate in the long-term treatment were excluded from the analyses. One of the 17 patients turned out to be a pseudo-responder and discontinued therapy at 12 months; however, it was not possible to exclude this patient from the analyses. ^n^ Ünal 2015: Type of PKU was unknown in 1 patient. Only 51 out of 75 responders were included: 21 patients who were not treated with protein substitute before BH4 were excluded, as well as 3 patients for whom BH4 treatment was stopped due to unsatisfactory metabolic control with little improvement in Phe tolerance (long-term non-responders). ^o^ Feldmann 2017: Out of 46 responders, 30 were included in the analyses: 35 patients completed the study but 5 patients who were not able to increase Phe tolerance after BH4 were excluded (long-term non-responders). ^p^ Rocha 2017: From a total of 13 responders, 9 were included: 4 patients either not taking any protein substitute before BH4 (*n* = 1 due to non-compliance, *n* = 1 not required), or with a follow-up duration less than 3 months (*n* = 1), or with unsatisfactory treatment results (*n* = 1 long-term non-responder) were excluded. ^q^ Evers 2018: From a total of 21 responders, 18 were included in the analyses: 2 patients with missing data on protein substitute intakes and 1 patient who was not treated with protein substitute before BH4 treatment were excluded. ^r^ Paras 2018: In this conference poster, the authors chose to only report on those patients who could be treated solely with BH4. From a total of 22 responders, only 8 were included: 13 patients who were not treated with protein substitutes before BH4 and 1 patient with maternal PKU were excluded.

**Table 3:** Quality appraisal and risk of bias.

Study (Author, Year)	Items of “Quality Assessment Tool for Before-After (Pre-Post) Studies with No Control Group”												Overall
	1	2	3	4	5	6	7	8	9	10	11	12	
Bélanger-Quintana 2005 [38]	x	+	?	?	+	+	+	NA	?	x	+	NA	Fair
Lambruschini 2005 [45]	+	+	?	+	+	+	+	NA	+	+	+	NA	Fair
Burlina 2009 [40]	+	+	?	?	+	+	+	NA	?	x	+	NA	Fair
Singh 2010 [49]	+	+	?	x	+	+	+	NA	+	+	+	NA	Fair
Vilaseca 2010 [51]	+	+	?	?	+	+	+	NA	?	x	+	NA	Fair
Singh 2011 [48] Douglas 2013a [42] Douglas 2013b [43] Brantley 2018 [39]	+	+	?	+	+	+	+	NA	+	+	+	NA	Good
Hennermann 2012 [17]	+	+	?	x	?	+	+	NA	+	x	+	NA	Fair
Leuret 2012 [46]	x	+	?	?	+	?	+	NA	+	+	?	NA	Fair
Aldámiz-Echevarría 2013 [36]	+	+	?	?	?	+	+	NA	?	x	+	NA	Fair
Demirdas 2013 [41]	+	+	?	x	?	?	x	NA	?	+	?	NA	Poor
Aldámiz-Echevarría 2015 [37]	+	+	?	?	?	+	+	NA	?	+	+	NA	Fair
Scala 2015 [47]	x	+	?	x	?	+	+	NA	+	+	+	NA	Fair
Thiele 2015 [29]	+	+	?	?	+	+	+	NA	?	+	+	NA	Fair
Ünal 2015 [50] Gökmen Özel 2014 [52]	+	+	+	?	+	+	+	NA	?	+	+	NA	Good
Feldmann 2017 [16]	+	+	?	x	?	+	+	NA	x	x	+	NA	Fair
Rocha 2017 [54]	+	x	?	?	+	?	+	NA	?	+	?	NA	Fair
Evers 2018 [44]	+	+	?	+	+	+	+	NA	?	+	+	NA	Good
Paras 2018 [53]	+	x	x	?	?	x	+	NA	?	x	?	NA	Poor

Each item was rated as low risk (“yes” = + ), unclear (“cannot determine/not reported” = ?), or high risk (“no” = x) for the following type of bias: objective study question (1); description of eligibility/selection criteria for the study population (2); representativeness of study population of general/clinical population of interest (3); selection bias (4); sample size, power, effect estimate (5); description of intervention, adherence, and deviations from intended interventions (6); measurement of outcomes (defined, valid, and reliable) (7); blinding of outcome assessors (8); loss to follow-up < 20% (9); statistical comparison for pre-to-post changes (10); frequency of repeated measurements (11); group-level interventions (12). NA, not applicable.

## Data Availability

Data is contained within the article or supplementary material.

## Supplementary Materials

The following are available online at https://www.mdpi.com/2072-6643/13/3/1040/s1: Figure S1: Change in phenylalanine intake (mg/kg/day) of long-term responders on BH4 treatment. Figure S2: Change in phenylalanine intake (mg/day) of long-term responders on BH4 treatment. Figure S3: Change in natural protein intake (g/kg/day) of long-term responders on BH4 treatment. Figure S4: Change in natural protein intake (g/day) of long-term responders on BH4 treatment. Figure S5: Change in protein equivalent intake from protein substitute (g/kg/day) of long-term responders on BH4 treatment. Figure S6: Change in protein equivalent intake from protein substitute (g/day) of long-term responders on BH4 treatment. Figure S7: Change in total protein intake (g/kg/day) of long-term responders on BH4 treatment. Figure S8: Change in total protein intake (g/day) of long-term responders on BH4 treatment. Table S1: Assessment and definition of BH4 responsiveness, long-term BH4 treatment, and protocol for adjusting dietary management. Table S2: Phenylalanine and protein intakes (total protein, natural protein, and protein equivalent from protein substitute) before and on BH4 treatment.
