# Supplementary material for: Protein Substitute Requirements of Patients with Phenylketonuria on BH4 Treatment: A Systematic Review and Meta-Analysis
Source: Nutrients. 2021 Mar 23;13(3):1040. doi: 10.3390/nu13031040 (PMC8004763; doi:10.3390/nu13031040)
Supplement: Supplementary file 1 [file nutrients-13-01040-s001.zip › SupplementaryFiles/20210218-SRBH4_SupplementaryMaterials.docx]

Supplementary materials

[Figure S1. Change in phenylalanine intake (mg/kg/day) of long-term responders on BH4 treatment. 2](#_Toc61463102)

[Figure S2. Change in phenylalanine intake (mg/day) of long-term responders on BH4 treatment. 2](#_Toc61463103)

[Figure S3. Change in natural protein intake (g/kg/day) of long-term responders on BH4 treatment. 3](#_Toc61463104)

[Figure S4. Change in natural protein intake (g/day) of long-term responders on BH4 treatment. 3](#_Toc61463105)

[Figure S5. Change in protein equivalent intake from protein substitute (g/kg/day) of long-term responders on BH4 treatment. 4](#_Toc61463106)

[Figure S6. Change in protein equivalent intake from protein substitute (g/day) of long-term responders on BH4 treatment. 4](#_Toc61463107)

[Figure S7. Change in total protein intake (g/kg/day) of long-term responders on BH4 treatment. 5](#_Toc61463108)

[Figure S8. Change in total protein intake (g/day) of long-term responders on BH4 treatment. 5](#_Toc61463109)

[Table S1. Assessment and definition of BH4 responsiveness, long-term BH4 treatment and protocol for adjusting dietary management. 6](#_Toc61463110)

[Table S2. Phenylalanine and protein intakes (total protein, natural protein and protein equivalent from protein substitute) before and on BH4 treatment. 12](#_Toc61463111)

**
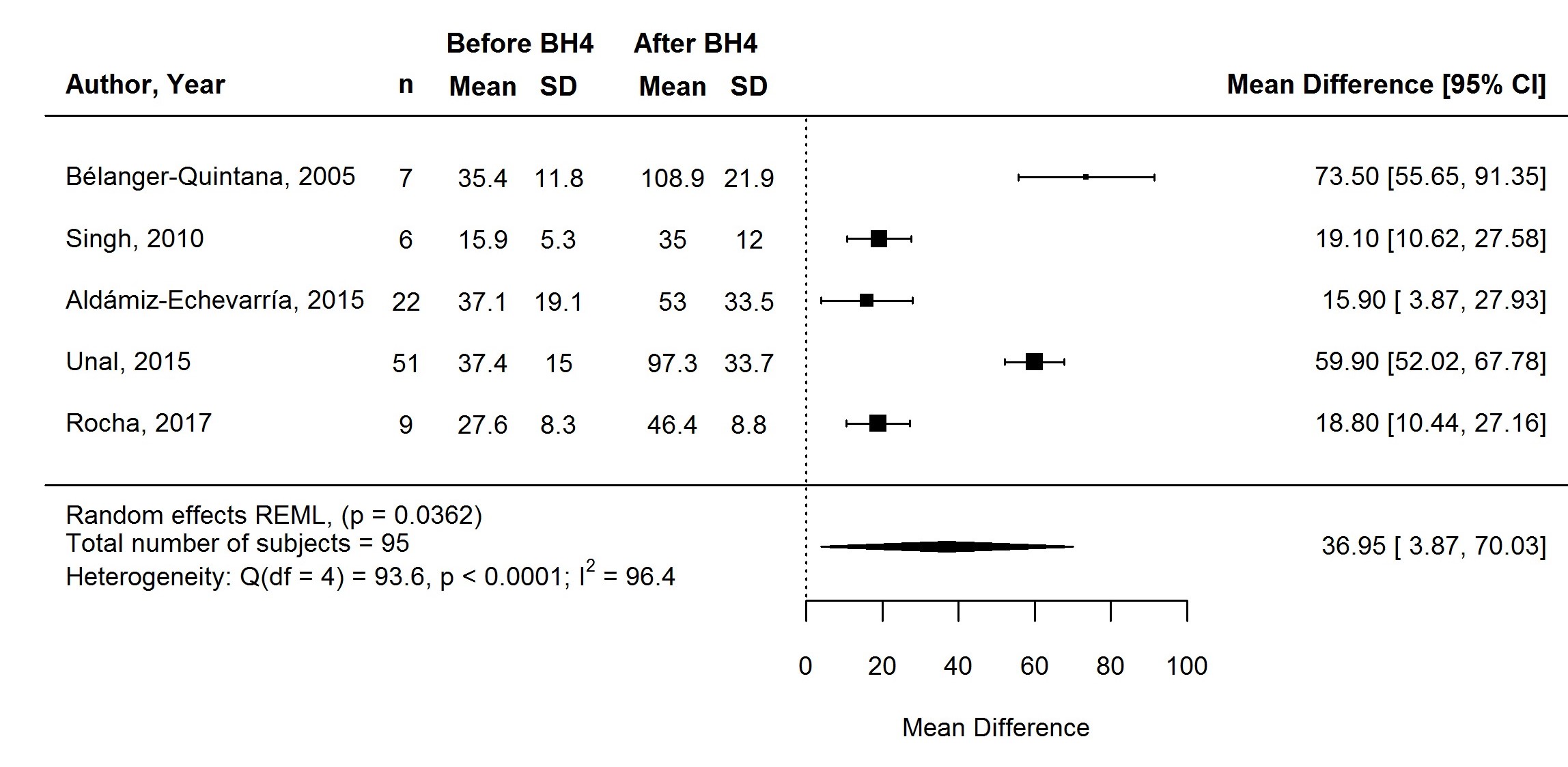
**

# Figure S1. Change in phenylalanine intake (mg/kg/day) of long-term responders on BH4 treatment.

Abbreviations: BH4, tetrahydrobiopterin; CI: confidence interval; n: sample size; SD: standard deviation.

**
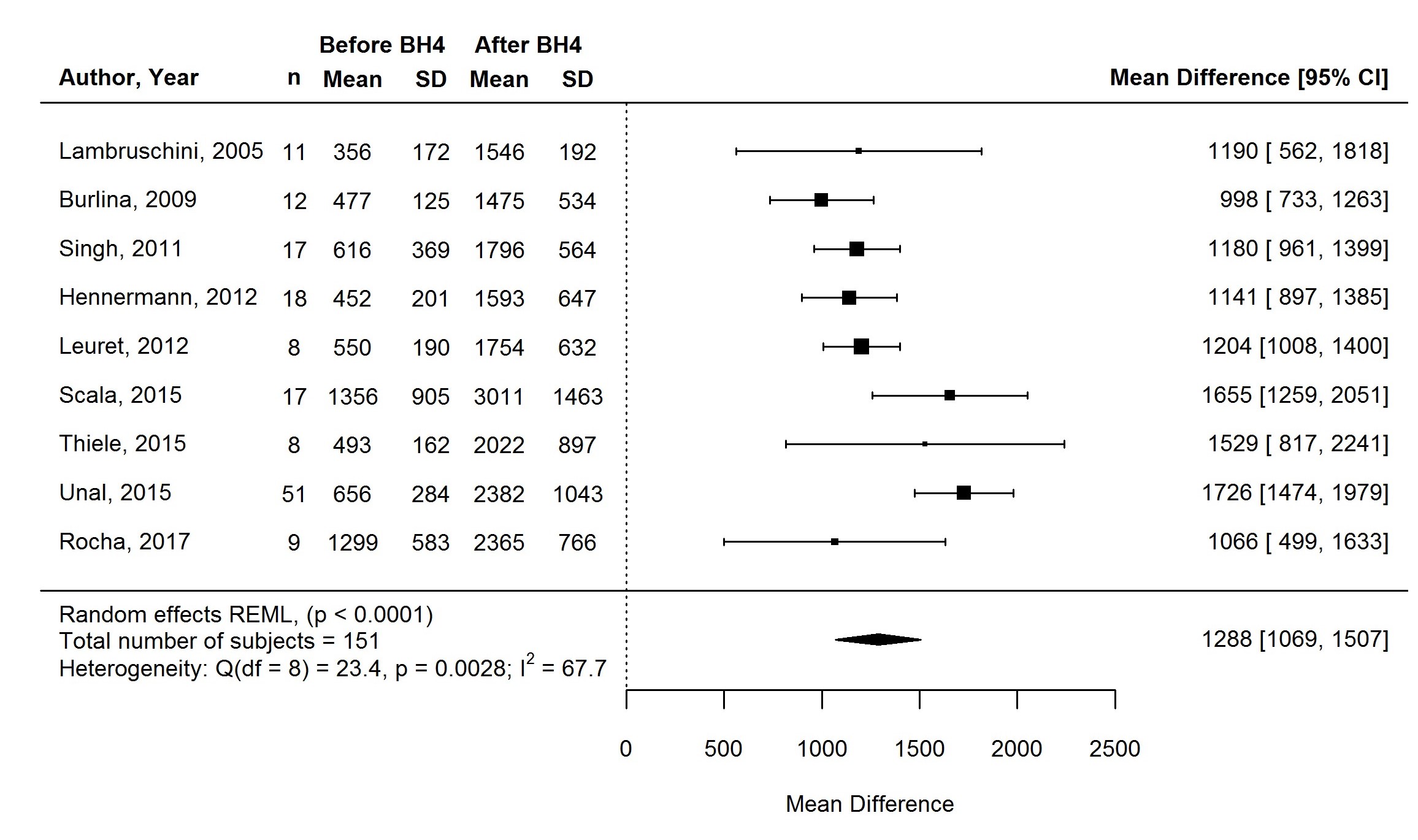
**

# Figure S2. Change in phenylalanine intake (mg/day) of long-term responders on BH4 treatment.

Abbreviations: BH4, tetrahydrobiopterin; CI: confidence interval; n: sample size; SD: standard deviation.

**
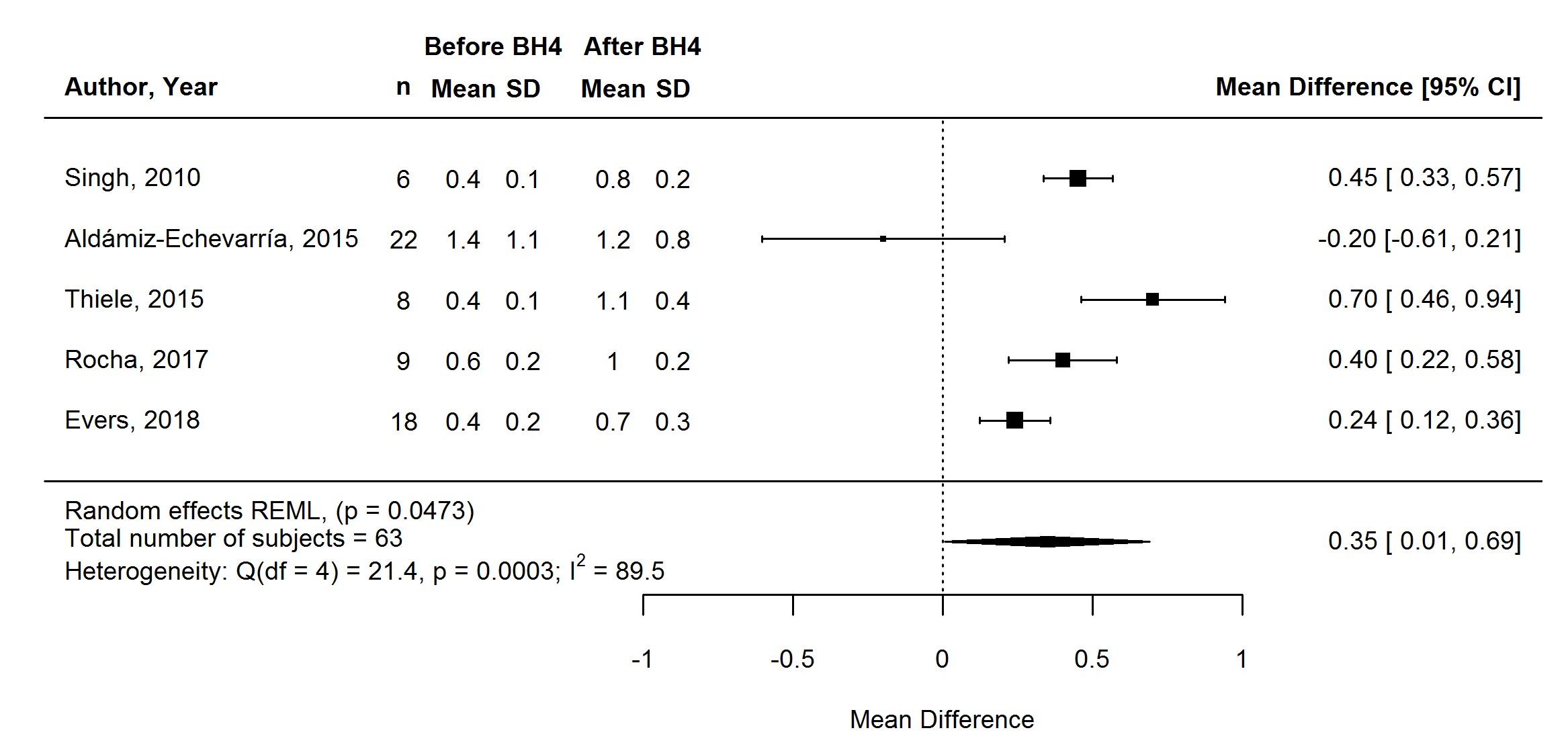
**

# Figure S3. Change in natural protein intake (g/kg/day) of long-term responders on BH4 treatment.

Abbreviations: BH4, tetrahydrobiopterin; CI: confidence interval; n: sample size; SD: standard deviation.

**
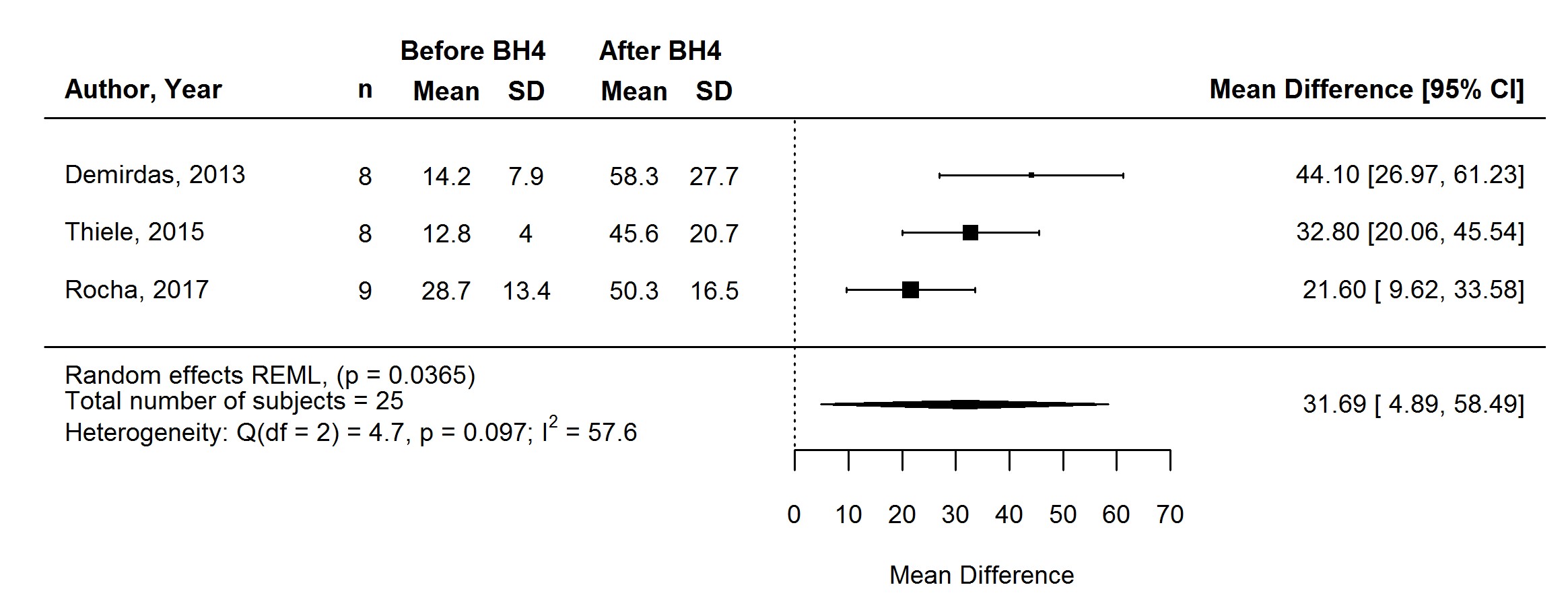
**

# Figure S4. Change in natural protein intake (g/day) of long-term responders on BH4 treatment.

Abbreviations: BH4, tetrahydrobiopterin; CI: confidence interval; n: sample size; SD: standard deviation.

**
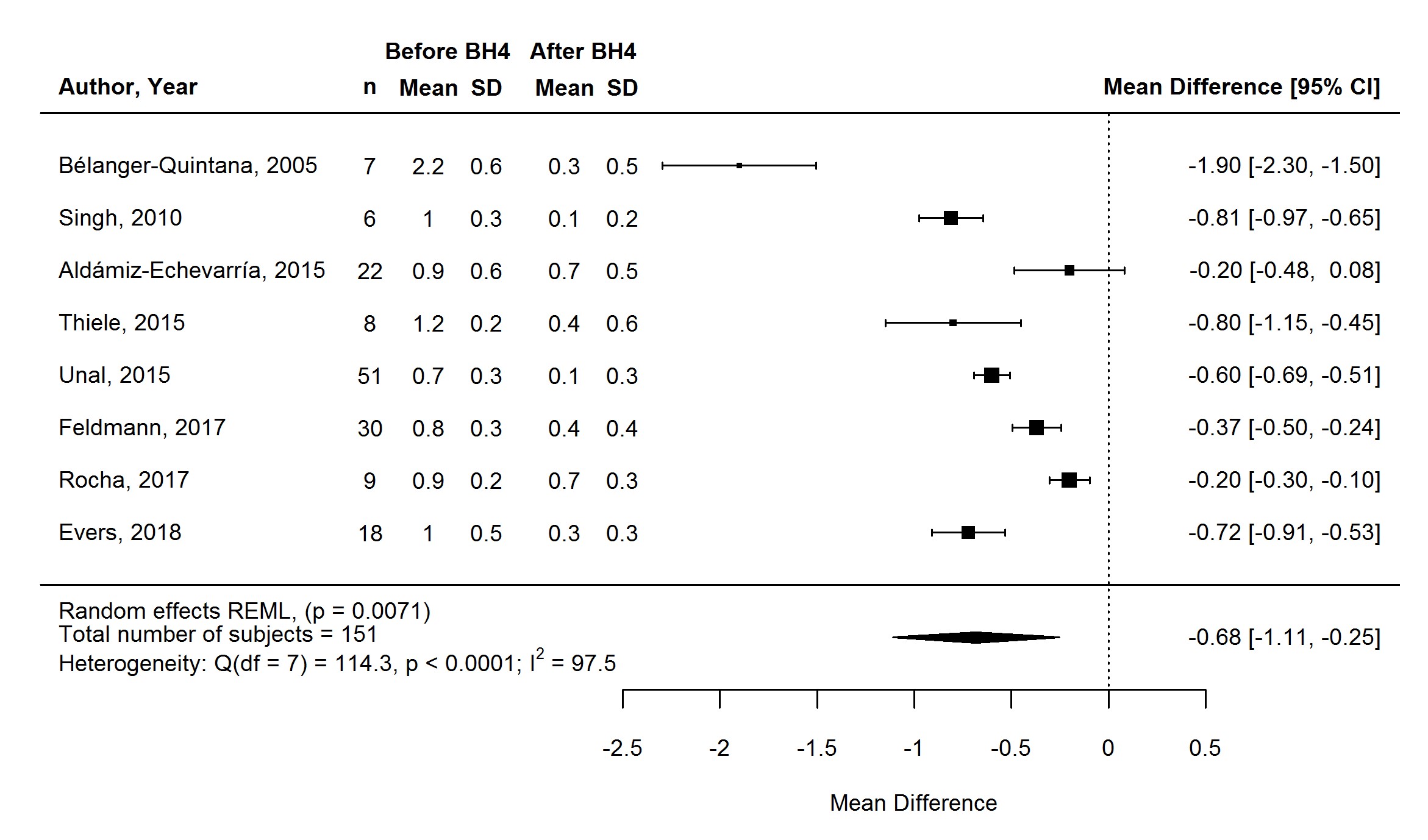
**

# Figure S5. Change in protein equivalent intake from protein substitute (g/kg/day) of long-term responders on BH4 treatment.

Abbreviations: BH4, tetrahydrobiopterin; CI: confidence interval; n: sample size; SD: standard deviation.

**
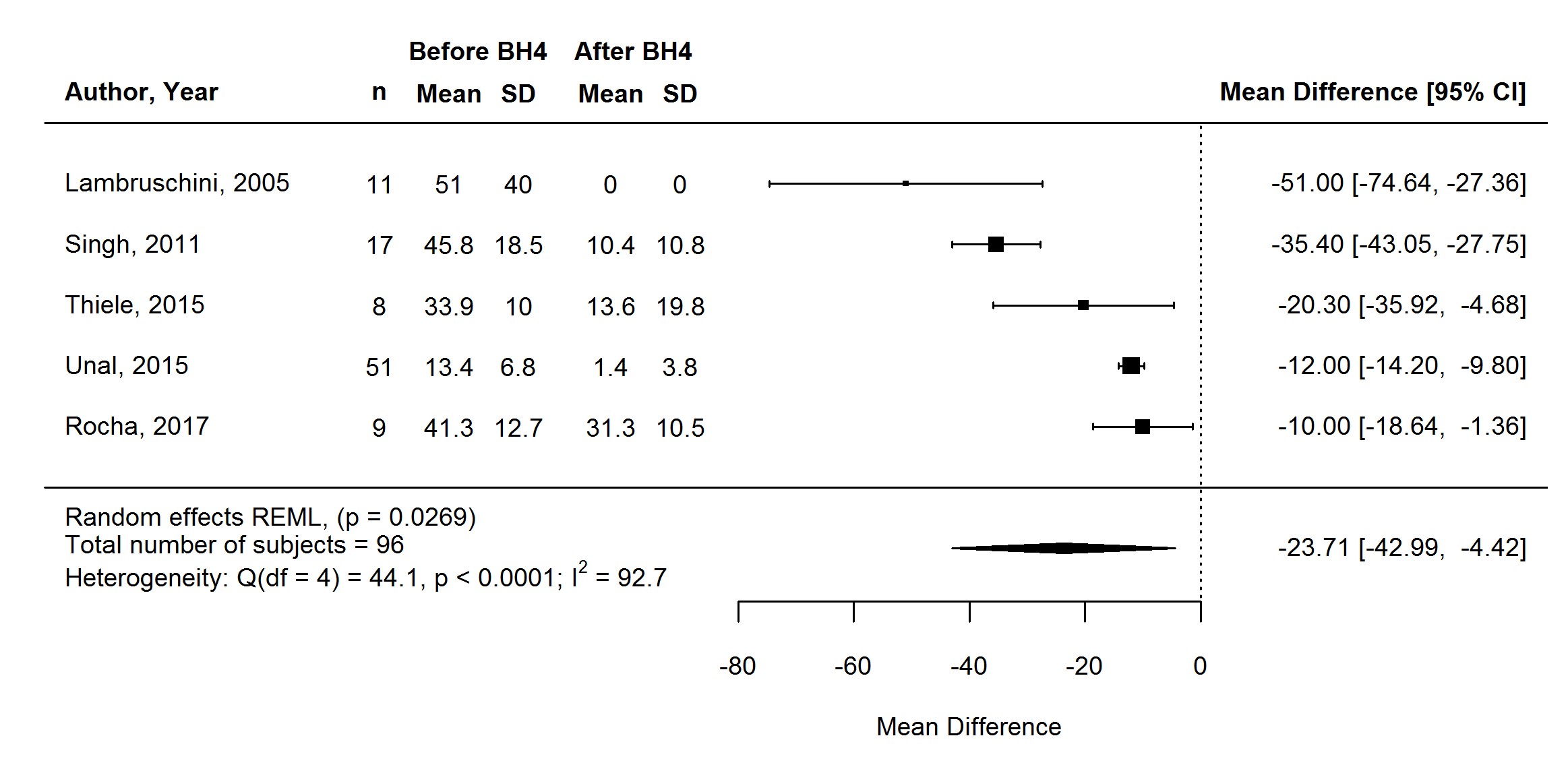
**

# Figure S6. Change in protein equivalent intake from protein substitute (g/day) of long-term responders on BH4 treatment.

Abbreviations: BH4, tetrahydrobiopterin; CI: confidence interval; n: sample size; SD: standard deviation.

**
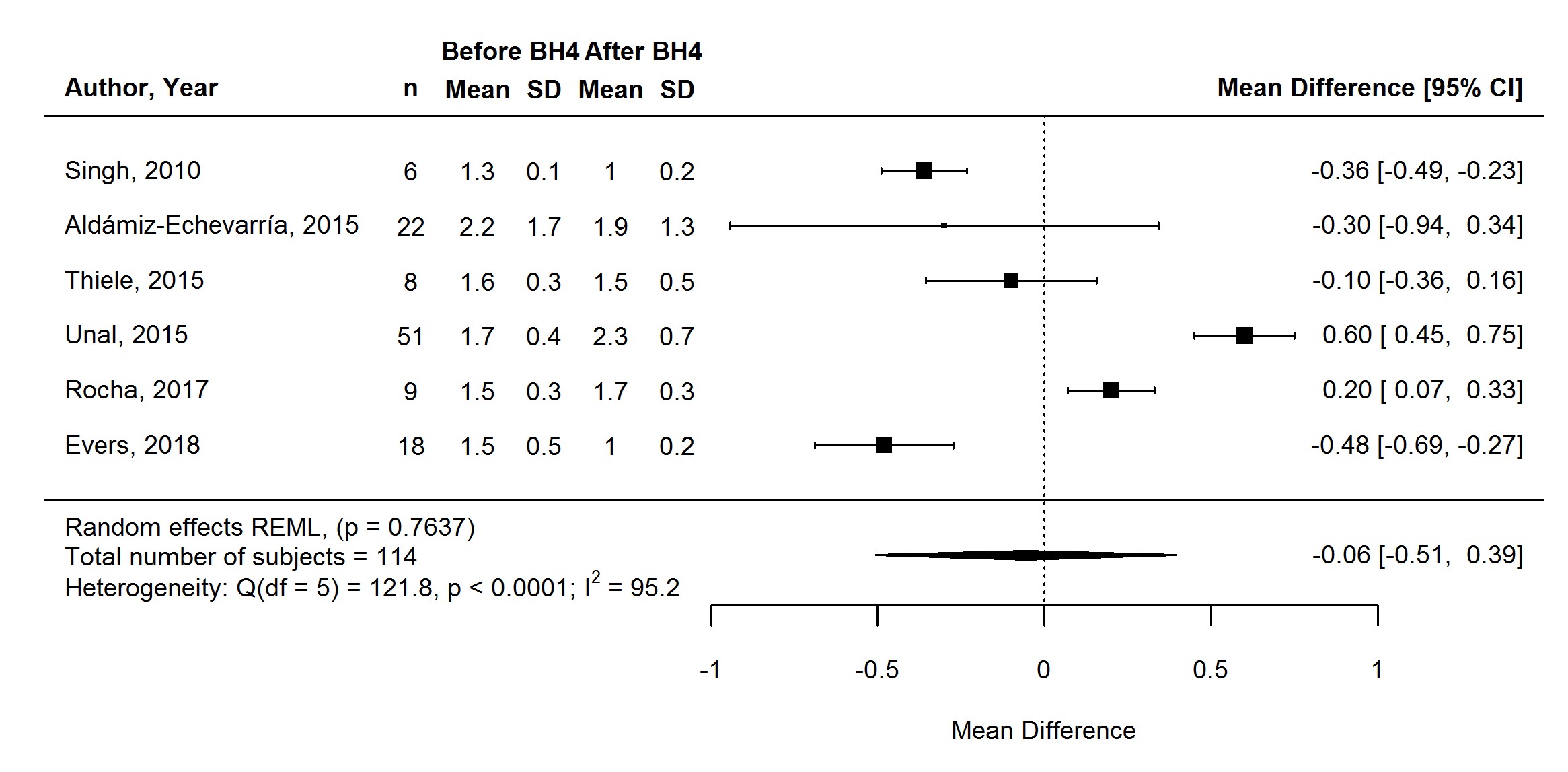
**

# Figure S7. Change in total protein intake (g/kg/day) of long-term responders on BH4 treatment.

Abbreviations: BH4, tetrahydrobiopterin; CI: confidence interval; n: sample size; SD: standard deviation.

**
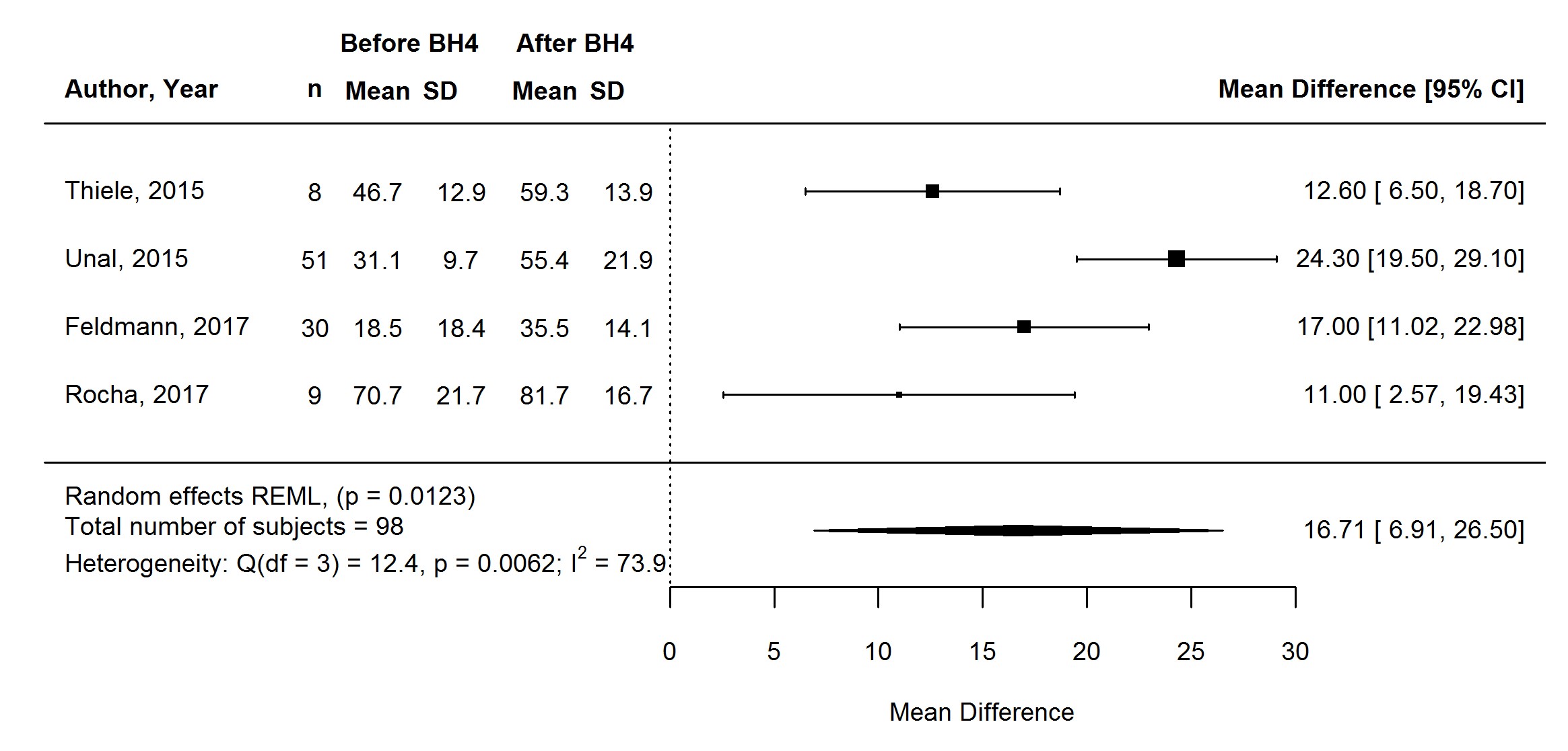
**

# Figure S8. Change in total protein intake (g/day) of long-term responders on BH4 treatment.

Abbreviations: BH4, tetrahydrobiopterin; CI: confidence interval; n: sample size; SD: standard deviation.

# Table S1. Assessment and definition of BH4 responsiveness, long-term BH4 treatment and protocol for adjusting dietary management.

| **Reference** | **Protocol for defining BH4 responsiveness and adjusting dietary management during long-term treatment** | **Long-term responders/non-responders ^1^** |
| --- | --- | --- |
| Bélanger-Quintana 2005 [38] | ***BH4 loading test:*** Two days prior to the test and during the entire testing period, patients had no dietary restrictions and interrupted their special protein supplements. A single dose of BH4 (20 mg/kg) was administered orally and blood filter spots were taken from 0 to 24 h after the challenge. Patients with Phe levels at diagnosis <360μmol/L (mHPA patients) were subjected to a single Phe load and a second combined Phe/BH4 challenge (100mg Phe 3h before BH4) in 2 consecutive days.  ***Definition of short-term responsiveness:*** Decrease in blood Phe >30% from baseline at 8h. Patients with a decrease >30% at 12-16h were considered slow responders.  ***Long-term treatment and protocol for adjusting dietary management:*** Initiated in 7 PKU responders who were on a Phe-restricted diet + PS (not in slow responders, not in mHPA).  The initial dose of BH4 was 10 mg/kg/day. The diet was completely liberalized, without Phe-free formula supplementation. Changes in the therapeutic approach were made over time on the basis of blood Phe levels. At the beginning of treatment, patients took 2–3 samples of Phe daily in blood filter spots. The number of samples was reduced as the levels of Phe were consistently in the therapeutic range, and the dosage of BH4 and the diet were considered optimal for each patient. The levels considered to be adequate varied with the patient’s age: <360μmol/L until 6 years of age, <540μmol/L from 6 to 9 years, and <630μmol/L from 9 years to adulthood.  ***Definition of long-term responsiveness:*** Not described. | 7/0 |
| Lambruschini 2005 [45] | ***Combined Phe/BH4 loading test:*** After at least 3h fasting, 100mg Phe/kg bodyweight was given orally. After 3h, a single dose of 20 mg BH4/kg bodyweight was administered orally. Blood samples for amino acid analysis were taken at 3h before Phe loading and at 0, 3, 7, 11, and 21 h after BH4 loading. ^2^  ***Definition of short-term responsiveness:*** Decrease in plasma Phe >30% from baseline at 21h.  ***Long-term treatment and protocol for adjusting dietary management:*** Initiated in 14 PKU patients with good response (45-94% decrease in plasma Phe) who were on a Phe-restricted diet + PS.  An initial dose of 5mg/kg/day was applied. BH4 was administered in 3 daily doses. Phe-restricted diet was progressively liberalised by adding 200mg Phe/day every week for two months, while PS was gradually reduced until complete removal was achieved. Patients were clinically and nutritionally evaluated monthly throughout the BH4 treatment.  ***Definition of long-term responsiveness:*** Increase in Phe tolerance >400mg Phe/day and complete removal of PS. BH4 therapy was discontinued otherwise. | **11/3** |
| Burlina 2009 [40] | ***BH4 loading test:*** Patients were instructed to continue the same dietary practice before and during the test. They were given 20mg BH4/kg after 3h of fasting and 30min before a meal to ensure good GI absorption. Blood sampling was done before and at 4, 8, 12, and 24h after BH4 administration.  ***Definition of short-term responsiveness:*** Decrease in plasma Phe ≥30% from baseline at 8h. Patients with a decrease <30% after 12-16h were considered partially responsive/slow responders.  ***Long-term treatment and protocol for adjusting dietary management:*** Initiated in 12 PKU responders with initial blood Phe level >450μmol/L and non-compliant with the Phe-restricted diet.  During the treatment the diet was relaxed according to the actual plasma Phe concentration. Regular follow-up blood samples were obtained before the first morning meal.  ***Definition of long-term responsiveness:*** Not described. | 12/0 |
|  |  |  |
| Singh 2010  [49] | ***BH4 loading test:***  One week of BH4 treatment at a dose of 20mg/kg/day.  ***Definition of short-term responsiveness:*** Decrease in plasma Phe ≥30% from baseline after 1 week.  ***Long-term treatment and protocol for adjusting dietary management:*** Initiated in 6 PKU responders who were on a Phe-restricted diet + PS.  Stage 1: Once responsiveness was established, change in Phe tolerance was determined. Responsive patients taking BH4 were progressively challenged with dry milk powder weekly until Phe concentrations exceeded 360μmol/L.  Stage 2: Once the maximum Phe tolerance was established using milk powder, PS intake was progressively decreased each week. PS amount was titrated using plasma Phe and serum transthyretin (prealbumin) concentrations as proxies of metabolic control and protein status, respectively. PS was reintroduced to the diet, as needed, in order to stabilize plasma phenylalanine <360μmol/ L and to keep serum transthyretin within normal limits (19–38mg/dl according to the reference range for the performing laboratory). After stabilizing the PS requirements of the patient, milk powder was displaced with regular foods as the source of intact protein. Adequacy of the diet was assessed using patient-recorded 3-day dietary records; dietary prescriptions were adjusted to ensure sufficient macro- and micronutrient intake.  ***Definition of long-term responsiveness:*** Not described. | 6/0 |
| Vilaseca 2010 [51] | ***Combined Phe/BH4 loading test:*** After at least 3h fasting, 100mg Phe/kg bodyweight was given orally. After 3h, a single dose of 20 mg BH4/kg bodyweight was administered orally. Blood samples for amino acid analysis were taken at 3h before Phe loading and at 0, 3, 7, 11, and 21h after BH4 loading.  ***Definition of short-term responsiveness:*** Decrease in plasma Phe >30% from baseline at 21h.  ***Long-term treatment and protocol for adjusting dietary management:*** Initiated 10 PKU responders who were on a Phe-restricted diet + PS. ^3^  An initial dose of 5mg/kg/day was applied to the selected PKU patients, but the BH4 dose was modified (5-15mg/kg/day) according to the individual patients' response, and PS was removed, so that BH4 was the only therapy in these patients. Natural proteins were allowed according to WHO recommendations for age and sex.  ***Definition of long-term responsiveness:*** Not described. | 10/0 |
| Singh 2011 [48]  Douglas 2013a [42]  Douglas 2013b [43]  Brantley 2018 [39] | ***BH4 loading test:*** One month of BH4 treatment at a dose of 20mg/kg/day.  ***Definition of short-term responsiveness:*** Decrease in plasma Phe ≥15% from baseline after 1 month.  ***Long-term treatment and protocol for adjusting dietary management:*** Initiated in 27 PKU responders. ^4^  --Patients restricting intact protein and having plasma Phe <360μmol/L after 1 month of BH4 were instructed to add 20g of non-fat dry milk powder (~350mg Phe or 6.8g protein) to their diet each week. After the new dietary Phe tolerance was established (quantity of dietary Phe consumed prior to blood Phe >360μmol/L), medical food intake was progressively decreased by 25% of baseline prescription each week. The patient's new medical food prescription was established as the intake associated with the last blood filter paper Phe concentration in the therapeutic range, ensuring dietary protein adequacy. Once dietary Phe and medical food tolerance were established, intact protein sources displaced milk powder in the diet. Female responders of childbearing potential were encouraged to maintain the taste for medical food by consuming a fraction of their baseline prescription al food, typically 25%, even if intact protein tolerance could meet the patient's RDA.  --Patients consuming medical food and plasma Phe >360μmol/L after 1 month of BH4 therapy were instructed to decrease dietary Phe intake by ~350mg/week (6.5-7g of intact protein). A patient's dietary Phe intake was decreased until blood Phe was in the therapeutic range. Medical food intake was then progressively decreased as described above. Patients consuming completely liberalized diets without medical food and whose blood Phe >360 μmol/L were instructed to decrease dietary Phe intake until metabolic control was achieved, with medical food progressively added back into the diet 25% at a time as needed to ensure dietary protein adequacy.  ***Definition of long-term responsiveness:*** A “definitive responder“ (i.e. long-term responder) was defined as a responder who could increase dietary Phe tolerance by ≥300mg/day (~6g of intact protein) and decrease medical food need by ≥25% while maintaining their blood Phe <360μmol/L and meeting their age- and sex-specific RDA for protein. Preliminary responders who could not increase their dietary Phe tolerance and decrease their medical food needs while maintaining metabolic control were classified as “provisional responders“ (i.e. long-term non-responders). Patients who electively ate diets rich in Phe (meeting RDA protein needs through intact protein) and had plasma Phe <360μmol/L after 1 month of BH4 had no need to have their diets liberalized and were considered definitive responders. If medical food was being consumed, its necessity was evaluated. | **18/9** |
| Hennermann 2012  [17] | ***Neonatal BH4 loading test:*** 24h (n=56), or 8h loading test (n=26).  ***Definition of short-term responsiveness:*** Decrease in plasma Phe ≥30% from baseline.  ***Long-term treatment and protocol for adjusting dietary management:*** Initiated in 23 PKU patients with plasma Phe >600μmol/L, compliant with dietary treatment, and with at least 2 of the following criteria: a mild PKU phenotype, a positive neonatal BH4 loading test, and/or at least 1 potential BH4 responsive mutation.  On BH4 treatment, Phe intake was increased weekly if Phe levels were within the recommended range according to the patient's age and the patient's individual range. In patients with a baseline Phe tolerance of ≤500mg/day, Phe intake was increased weekly by 50mg/day; in patients with a baseline Phe tolerance of >500 mg/day, Phe intake was increased weekly by 100mg/day. Increase of dietary Phe intake was performed in 5 consecutive steps: 1. Low protein bread was replaced by “normal” bread. 2. Low protein pasta was replaced by “normal” pasta. 3. Milk and dairy products were included. 4. Meat and sausages were included. 5. Additionally, fish and eggs were included in the diet schedule. The intake of the amino acid formula was adapted weekly according to the actual individual Phe intake. In patients with a sufficient intake of natural protein by diet, amino acid formula was stopped. BH4 was started in a dose of 20mg/kg/day in all patients. In patients who could stop Phe-restricted diet and amino acid supplementation, BH4 was reduced slowly, in steps of 100mg/day weekly to 2-weekly, until reaching a final dose of 5-10mg/kg/day. In patients who still received small amounts of amino acid formula, BH4 was slowly titrated by not adapting BH4 doses to the increasing weight. However, BH4 doses were only reduced if Phe levels were kept within the recommended range according to the patient's age and the patient's individual range.  ***Definition of long-term responsiveness:*** Increase in Phe tolerance ≥2.5-fold. BH4 treatment was discontinued if Phe increase was <2.0-fold after a period of 3 months and <2.5-fold after a period of 4 months, if Phe plasma levels increased above the German recommended levels, and/or if patients were incompliant to BH4 treatment. | **18/5** |
| Leuret 2012 [46] | ***BH4 loading test:*** 24h loading test using a single oral dose of 20mg/kg. Neonatal testing for n=11; for n=4, test performed 3 days after starting an oral Phe load designed to increase basal Phe prior to treatment. The Phe load was performed by increasing either the natural protein intake (3 g/kg/day) or the Phe intake alone (Phe capsules, 100 mg/kg/day) and was maintained throughout the 24-hr BH4 loading test.  ***Definition of short-term responsiveness:*** Decrease in plasma Phe ≥30% from baseline during test.  Protocol for adjusting dietary management not described.  ***Long-term treatment and protocol for adjusting dietary management:*** Initiated in 8 PKU responders with poor compliance and unsatisfactory metabolic control with diet alone. ^5^  Protocol for adjusting dietary management not described.  ***Definition of long-term responsiveness:*** Not described. | 8/0 |
| Aldámiz-Echevarría 2013 [36] | ***BH4 loading test:*** 24h loading test at most hospitals, where patients were instructed to maintain their usual dietary habits before and during the test. At 1 hospital after 2005, 2-stage loading test: 24h or if patients did not respond within 24h, 1 week with a BH4 dose of 20 mg/kg/day and a daily protein intake meeting patients' age- and sex-specific RDAs.  ***Definition of short-term responsiveness:*** Most hospitals: Decrease in blood Phe ≥30% from baseline after 8h (primary/fast responders) or after 12h (late responders). At 1 hospital after 2005: Decrease in blood Phe ≥50% from baseline after 24h (fast responders) or Phe level remaining below a defined threshold (<360μmol/L for individuals <6y; <480μmol/L for those 6 to 10y, and <600μmol/L for those >10y) after 1 week (late responders).  ***Long-term treatment and protocol for adjusting dietary management:*** Initiated in responders (both fast and late) who were on a Phe-restricted diet + PS: 36 responders with a 2y follow-up and 10 with a 5y follow-up. ^6^  Responders were prescribed BH4 treatment, with a dose ranging from 5 to 20 mg/kg/day and a natural protein intake meeting RDAs. They gradually increased the intake of protein-rich foods, and consequently reduced the intake of protein in amino acid mixtures, in an attempt to enable them to resume a normal diet. Patients with Phe reduction <50% in BH4 loading test could not maintain an exclusively normal diet, but they obtained at least 70% of total protein from natural sources.  ***Definition of long-term responsiveness:*** Not described. | Patients with 2y follow-up:  36/0  Patients with 5y follow-up:  10/0 |
| Demirdas 2013 [41] | ***BH4 loading test:*** 48h loading test with an oral dose of 20 mg/kg once daily.  ***Definition of short-term responsiveness:*** Decrease in Phe ≥30% from baseline during test.  ***Long-term treatment and protocol for adjusting dietary management:*** Initiated in 10 who were on a Phe-restricted diet + PS. ^7^  Protocol for adjusting dietary management not described.  ***Definition of long-term responsiveness:*** Not described. |  |
| Aldámiz-Echevarría 2015 [37] | ***BH4 loading test:*** 24h loading test at most hospitals, where patients were instructed to maintain their usual dietary habits before and during the test. At 1 hospital after 2005, 2-stage loading test: 24h or if patients did not respond within 24h, 1 week with a BH4 dose of 20 mg/kg/day and a daily protein intake meeting patients' age- and sex-specific RDAs.  ***Definition of short-term responsiveness:*** Most hospitals: Decrease in blood Phe ≥30% from baseline after 8h (primary/fast responders) or after 12h (late responders). At 1 hospital after 2005: Decrease in blood Phe ≥50% from baseline after 24h (fast responders) or Phe level remaining <360μmol/L after 1 week (late responders).  ***Long-term treatment and protocol for adjusting dietary management:*** Initiated in 22 responders (both fast and late) who were on a Phe-restricted diet + PS.  In an attempt to enable patients to resume a normal diet, they gradually increased their intake of high biological value proteins such as milk and dairy products for several months, as well as foods with a moderate protein content (such as cereals), while maintaining the BH4 treatment, and consequently they reduced their intake of protein in amino acid mixtures.  ***Definition of long-term responsiveness:*** Not described. | 22/0 |
| Scala 2015 [47] | ***Pre-loading test protocol:*** Each patient was asked to comply to the assigned diet with Phe amounts corresponding to the known Phe tolerance and equally distributed in three main meals. Patients were asked to fill a meal diary, reviewed by the dietician. The dietary regimen had to be followed for at least 2 weeks before the BH4 loading test. In the week before the BH4 loading test, blood Phe and tyrosine were analyzed twice a week after an overnight fast to verify the compliance to diet on the basis of Phe values. Only a 15% variance between the two Phe values was accepted. If plasma Phe resulted ≥ 400μmol/L, the BH4 loading test was started; if plasma Phe resulted <400μmol/L, dietary Phe intake was increased every 10-15 days until blood Phe resulted ≥ 400μmol/L.  ***BH4 loading test:*** A 48h-long BH4 loading test was performed with BH4 tablets in 2 oral doses of 20mg/kg at T0 and T24h. Plasma Phe and Tyr were analyzed at 0, 4, 8, 12, 24, 32 and 48h. Patients were hospitalized during the loading test to ensure optimal compliance.  ***Definition of short-term responsiveness:*** Decrease in blood Phe >30% from baseline within 24h (rapid responders) or between T24 and T48h (slow responders).  ***Long-term treatment and protocol for adjusting dietary management:*** Initiated in 17 mHPA and PKU responders who were on a Phe-restricted diet + PS.  Responders started a long-term treatment with BH4 at the initial dose of 10 mg/kg/day. In some patients, the dose was changed based on the metabolic control. During the trial, all patients assumed 50 mg BH4 tablets, divided in 3 oral doses before meals, until September 2009, when therapy was shifted to sapropterin. Phe intake was increased by standard food exchange lists ranging from 75 to 225 mg/day, every 15-30 days if Phe levels resulted within the target. Plasma Phe was always dosed after the overnight fast. The reference Phe values in different age groups were 120-360μmol/L until 12 years of age and 120-600μmol/L for older ages. Outcome measures were improvement of metabolic control and of tolerance to Phe. Median plasma Phe obtained during the 5 years preceding BH4 treatment was used as index of metabolic control before BH4 and compared with plasma Phe levels obtained during BH4 therapy. Dietary Phe intake (mg/day) was assessed before starting and during BH4 therapy. A 3 days meal diary was analyzed at each visit.  ***Definition of long-term responsiveness:*** Significant increase of Phe intake compared to the historical tolerance and before BH4. | **16/1** |
|  |  |  |
| Thiele 2015 [29] | ***BH4 loading test:*** 6-week loading test. Period 1 (days -3 to 0): evaluation of the current classical treatment. The Phe tolerance was determined at study entry with the help of 3 nutrition protocols on 3 consecutive days. Period 2 (days 1-14): patients were instructed to double their daily Phe intake from natural protein, while the rest of the treatment (supply of Phe-free amino acid mixture) remained unchanged. The patients were asked to perform nutrition protocols 3 times a week and to collect a capillary blood sample for determination of Phe concentration on each day following a nutrition protocol. Period 3 (days 14-42): patients received 20 mg/kg/day of BH4, taken as a single oral dose in the morning, while continuing the increased Phe intake. Again, the patients performed nutrition protocols 3 times a week and collected blood samples always on the following day.  ***Definition of short-term responsiveness:*** Decrease in blood Phe >30% from baseline and/or Phe concentrations remaining within the therapeutic range when Phe consumption increased by 100%.  ***Long-term treatment and protocol for adjusting dietary management:*** Initiated in 8 mHPA and PKU responders who were on a Phe-restricted diet + PS.  Initial median BH4 dosage of 18mg/kg/day (range 10-19mg/kg/day). Patients received individual diet counseling at each clinical visit, at least 4 times a year. Liberalization of the diet (and change in PS intake) depended on adequacy of protein intake from natural food.  ***Definition of long-term responsiveness:*** Not described. | 8/0 |
| Ünal 2015 [50]  Gökmen Özel 2014 [52] | ***BH4 loading test:*** 48h loading test with a BH4 dose of 20 mg/kg/day. Phe powder was added to the diet and continued throughout the 48h BH4 loading test period to keep blood Phe levels high enough to be eligible for the test.  ***Definition of short-term responsiveness:*** Decrease in blood Phe >30% from baseline during test.  ***Long-term treatment and protocol for adjusting dietary management:*** Initiated in 54 mHPA and PKU responders who were on a Phe-restricted diet + PS. ^8^  Sapropterin treatment was initiated as a dose of 20mg/kg/day in all patients. Actual Phe tolerance was calculated in each patient before the administration of sapropterin. Dietary Phe intake was increased in increments of approximately 10mg/kg on a weekly basis (max 300-350 mg/day) during the initiation and adjustment phase. Initially, vegetable protein (ordinary bread, pasta, rice, etc.) was given. Animal protein was introduced thereafter, and the amount of Phe-free amino acid mixture was gradually decreased. If the patient had satisfactory metabolic control, the total initial BH4 dosage was not changed, with the dose per kilogram decreasing as long as the child gained weight. Once the dietary Phe introduction phase was completed, sapropterin was reduced to the dosage that was sufficient to maintain blood Phe levels within normal limits.  ***Definition of long-term responsiveness:*** Increase in Phe tolerance with satisfactory metabolic control. | **51/3** |
| Feldmann 2017 [16] | ***BH4 loading test:*** Sapropterin was initially administered at a dose of 10mg/kg/day at the time of the outpatient visit. This dose was repeated at home every 24 hours for another six days. On day 8, and regardless of the patient’s response to sapropterin, the sapropterin dose was increased to 20mg/kg/day for another seven days to test the individual’s response to the higher dose. The dietary regimen of the patients remained unchanged throughout the 2 weeks of the response test. Serum Phe levels were measured at 24 hours and seven days after the first dose of sapropterin and 24 hours and seven days after the increased dose.  ***Definition of short-term responsiveness:*** Decrease in serum Phe ≥30% from baseline on day 15.  ***Long-term treatment and protocol for adjusting dietary management:*** Initiated in 35 PKU responders who were on a Phe-restricted diet + PS. ^9^  Sapropterin was administered at a dose of 20mg/kg/day regardless of whether any response had occurred at 10 or 20mg/kg/day. To determine the responsive patients’ individual increase in Phe tolerance due to sapropterin treatment, the dietary Phe content was gradually increased over the following 6 weeks by the addition of natural protein in the diet, for example natural food like ordinary bread or pasta products. The amount of Phe-free amino acid supplements required was reduced as appropriate. The Phe levels in all patients were monitored weekly and were maintained within the age-appropriate target levels.  ***Definition of long-term responsiveness:*** Increase in Phe tolerance with satisfactory metabolic control. | **30/5** |
|  |  |  |
| Rocha 2017 [54] | ***BH4 loading test:*** One day monitoring, followed by a 48h responsiveness protocol with blood spots taken at T=-24, -16, -8, and T=0, 8, 16, 24, 32, 40 and 48h post BH4. Day 1: stable diet (similar as before) and 3 blood spots are taken (T=-24, -16 and -8h); Day 2: stable diet (similar as before) but 20 mg/kg BH4 is taken together with the breakfast (T=0) and 3 blood spots are taken (T=0 (fasted), 8, and 16h); Day 3: stable diet (similar as before) but 20 mg/kg BH4 is taken together with the breakfast (T=24h) and 3 blood spots are taken (T=24 (fasted), 32, 40h); Day 4: Blood spot taken fasted at T=48h.  ***Definition of short-term responsiveness:*** Decrease in blood Phe ≥30% from baseline during test.  ***Long-term treatment and protocol for adjusting dietary management:*** Initiated in 10 PKU responders who were on a Phe-restricted diet + PS. ^10^  Protocol for adjusting dietary management not described.  ***Definition of long-term responsiveness:*** Substantial improvement in Phe tolerance/natural protein intake and metabolic control. | **9/1** |
| Evers 2018 [44] | ***BH4 loading test:*** 48h loading test with a single daily dose (20mg/kg/day) on 2 consecutive days.  ***Definition of short-term responsiveness:*** Decrease in blood Phe >30% from baseline during test.  ***Long-term treatment and protocol for adjusting dietary management:*** Initiated in 18 PKU responders who were on a Phe-restricted diet + PS. ^11^  All patients were regularly seen by a dietician, thereby managing changes in diet following BH4 treatment. Further details not described.  ***Definition of long-term responsiveness:*** Not described. | 18/0 |
| Paras 2018 [53] | ***BH4 loading test:***  Not described.  ***Definition of short-term responsiveness:*** Not described.  ***Long-term treatment and protocol for adjusting dietary management:*** Initiated in 8 responders who were on a Phe-restricted diet + PS. ^12^  Protocol for adjusting dietary management not described.  ***Definition of long-term responsiveness:*** Not described. | 8/0 |

Abbreviations: (6R-)BH4: tetrahydrobiopterin; mHPA: mild hyperphenylalaninemia; PKU: phenylketonuria; Phe: phenylalanine; PS: protein substitute; RDA: Recommended Dietary Allowance; n/a: not available.

^1^ Only long-term responders (who were on a Phe-restricted diet + PS before BH4) were included in our analyses. Some papers described patients who turned out not to be responders in the long term (indicated in bold). Other authors did not mention this; however, given the duration of follow-up, it was assumed that all responders described were (‘true’) long-term responders.

^2^ Lambruschini 2005: Test performed in 66/73 patients. The table does not describe the 24h BH4 loading test performed in 7 neonates before starting the Phe-restricted diet, as these patients were not included in our analyses

^3^ Vilaseca 2010: BH4 treatment also initiated in 3 neonates, before starting the Phe-restricted diet. These 3 patients were excluded from our analyses.

^4^ Singh 2011: A total of 32 patients were found to be preliminary responders but 5 were not included: 2 were non-compliant and thus not classified for long-term responsiveness, 1 was taken off BH4 early, 1 was lost to follow-up and 1 was not taking PS before BH4 treatment. Note that one additional patient was lost to follow-up between 4mo (n=18) and 1y (so n=17).

^5^ Leuret 2012: BH4 treatment also initiated in 7 neonates, before starting the Phe-restricted diet. These 7 patients were excluded from our analyses.

^6^ Aldámiz-Echevarría 2013: Unclear if patients with a 5y follow-up were also described in the group of patients with a 2y follow-up. It was assumed that the 2 cohorts comprised different patients.

^7^ Demirdas 2013: Two additional responders for whom PS intake was unknown not included.

^8^ Ünal 2015: Twenty-one additional responders not taking any PS before BH4 treatment not included.

^9^ Feldmann 2017: Only 35/46 responders completed the study and were included.

^10^ Rocha 2017: Three additional responders not included: 1 no need for PS, 1 not taking any PS due to non-compliance, 1 follow-up <3 months.

^11^ Evers 2018: Three additional responders not included: 2 missing data on PS intake and 1 not treated with PS before BH4 treatment.

^12^ Paras 2018: Fourteen additional responders not included: 12 not treated with PS before BH4 treatment and 1 patient with maternal PKU.

# Table S2. Phenylalanine and protein intakes (total protein, natural protein and protein equivalent from protein substitute) before and on BH4 treatment.

| **Reference** | **Duration** | **Phe intake** | | **Total protein intake** | | **Natural protein intake** | | **Protein intake from PS** | |
| --- | --- | --- | --- | --- | --- | --- | --- | --- | --- |
|  | **on BH4 (mo)** | **before BH4** | **on BH4** | **before BH4** | **on BH4** | **before BH4** | **on BH4** | **before BH4** | **on BH4** |
| Bélanger-Quintana 2005 [38] | *Mean (SD, range)* | *Mean (SD); mg/kg/day* | | n/a | | n/a | | *Mean (SD); g/kg/day* | |
|  | 11.3 (6.5, 5.0-18.0) | 35.4 (11.8) | 108.9 (21.9) |  | |  | | 2.2 (0.6) | 0.3 (0.5) |
|  |  | *Median (range); mg/kg/day* | |  | |  | | *Median (range); g/kg/day* | |
|  |  | 44.0 (20.0-45.0) | 111.0 (75.0-133.0) |  | |  | | 2.0 (1.5-3.0) | 0.0 (0.0-1.0) |
| Lambruschini 2005  [45] | 12.0 | *Mean (SD, range); mg/day* | | n/a | | n/a | | *Mean (SD); g/day* | |
|  |  | 356.0 (172.0, 201.0-600.0) | 1546.0 (192.0, 1240.0-1801.0) |  | |  | | 51.0 (40.0) | 0.0 (0.0) |
| Burlina 2009 [40] | *Mean (SD, range)* | *Mean (SD); mg/day* | | n/a | | n/a | | *Mean (SD); g/day* | |
|  | 42.0 (21.6, 6.0-84.0) | 476.7 (125.4) | 1475.0 (534.5) |  | |  | | n/a | 0.0 (0.0) |
|  |  | *Median (range); mg/day* | |  | |  | |  |  |
|  |  | 450.0 (350.0-700.0) | 1400.0 (800.0-2700.0) |  | |  | |  |  |
| Singh 2010  [49]^a^ | 24.0 | *Mean (SD); mg/kg/day* | | *Mean (SD); g/kg/day* | | *Mean; g/kg/day* | | *Mean; g/kg/day* | |
|  |  | 15.9 (5.3) | At 3mo: 34 (13.8) | 1.3 (0.1) | At 3mo: 1.0 (0.2) | 0.4 | At 3mo: 0.8 | 1.0 | At 3mo: 0.2 |
|  |  |  | At 12mo: 36 (5.0) |  | At 12mo: 1.1 (0.1) |  | At 12mo: 0.8 |  | At 12mo: 0.3 |
|  |  |  | At 24mo: 35 (12.0) |  | At 24mo: 1.0 (0.2) |  | At 24mo: 0.8 |  | At 24mo: 0.2 |
| Vilaseca 2010 [51] | *Mean (SD, range)* | *Mean (SD); mg/kg/day* | | n/a | | n/a | | *Mean (SD); g/day* | |
|  | 68.4 (2.4, 63.6-72.0) | n/a | 57.5 (21.5) |  | |  | | n/a | 0.0 (0.0) |
|  |  | *Median (range); mg/kg/day* | |  | |  | |  |  |
|  |  | n/a | 51.3 (34.8-93.5) |  | |  | |  |  |
| Singh 2011  [48]^b^  Douglas 2013a [42]  Douglas 2013b [43]  Brantley 2018 [39] | 12.0 | *Mean (SD); mg/day* | | n/a | | n/a | | *Mean (SD); g/day* | |
|  |  | 704.0 (518.0) | At 4mo: 1922.0 (612.0) |  | |  | | 43.3 (20.3) | At 4mo: 7.8 (10.5) |
|  |  | 616.1 (368.7) | At 12mo: 1796.0 (563.8) |  | |  | | 45.8 (18.5) | At 12mo: 10.4 (10.8) |
|  |  | *Median (IQR); mg/day* | |  | |  | | *Median (IQR); g/day* | |
|  |  | 791.0 (529.0-2207.0) | At 12mo: 1198.0 (993.0-1457.0) |  | |  | | n/a | At 12mo: 0.0 (0.0) |
| Hennermann 2012  [17] | *Mean (SD, range)* | *Mean (SD); mg/day* | |  | |  | | *Mean (SD); g/kg/day* | |
|  | 48.0 (27.0, 8.0-105.0) | 452.0 (201.0) | 1593.0 (647.0) | n/a | | n/a | | n/a | 0.3 (0.4) |
|  |  | *Median (range); mg/day* | |  | |  | | *Median (range); g/kg/day* | |
|  |  | 417.0 (190.0–960.0) | 1700.0 (650.0–2600.0) |  | |  | | n/a | 0.3 (0-0.9) |
| Leuret 2012 [46] | *Median (range)* | *Mean (SD); mg/day* | | n/a | | n/a | | n/a | |
|  | 23.0 (7.0-80.0)^c^ | 550.0 (190.0) | 1754.0 (632.0) |  | |  | |  | |
| Aldámiz-Echevarría 2013  [36] |  | *Median (IQR); mg/kg/day* | | *Median (IQR); g/kg/day* | | *Median (IQR); g/kg/day* | | *Median (IQR); g/kg/day* | |
|  | 24.0 (cohort 1)^d^ | 29.9 (18.3-52.3) | 41.2 (22.9-48.9) | 1.8 (1.0-3.6) | 1.5 (0.7-2.2) | 0.7 (0.4-1.1) | 0.8 (0.5-1.0) | 0.9 (0.7-1.2) | 0.5 (0.2-1.3) |
|  | 60.0 (cohort 2)^d^ | 30.8 (24.6-54.8) | 38.1 (17.6-47.9) | 1.7 (1.3-2.2) | 1.2 (0.7-1.7) | 0.8 (0.3-1.6) | 0.9 (0.7-1.1) | 0.7 (0.5-1.2) | 0.3 (0.0-0.7) |
| Demirdas 2013 [41] | 17.0-24.0 | n/a | | n/a | | *Mean (SD); g/day* | | n/a | |
|  |  |  | |  | | 14.2 (7.9) | 58.3 (27.7) |  | |
| Aldámiz-Echevarría 2015 [37] | 12.0 | *Mean (SD); mg/kg/day* | | *Mean (SD); g/kg/day* | | *Mean (SD); g/kg/day* | | *Mean (SD); g/kg/day* | |
|  |  | 37.1 (19.1) | 53.0 (33.5) | 2.2 (1.7) | 1.9 (1.3) | 1.4 (1.1) | 1.2 (0.8) | 0.9 (0.6) | 0.7 (0.5) |
| Scala 2015  [47] | *Mean (SD, range)* | *Mean (SD); mg/day* | | n/a | | n/a | | n/a | |
|  | 68.8 (17.2, 12.0-84.0) | 1355.9 (905.2) | 3010.6 (1463.4) |  | |  | |  | |
|  |  | *Median (range); mg/day* | |  | |  | |  | |
|  |  | 1228.0  (280.0-3187.0) | 3000.0  (1010.0-5000.0) |  | |  | |  | |
| Thiele 2015 [29] | 24.0 | *Mean (SD); mg/day* | | *Mean (SD); g/day* | | *Mean (SD); g/day* | | *Mean (SD); g/day* | |
|  |  | 493.2 (161.8) | At 3mo: 2208.9 (1336.4) | 46.7 (12.9) | At 3mo: 56.0 (24.2) | 12.8 (4.0) | At 3mo: 48.2 (29.8) | 33.9 (10.0) | At 3mo: 7.8 (14.6) |
|  |  |  | At 24mo: 2021.9 (897.4) |  | At 24mo: 59.3 (13.9) |  | At 24mo: 45.6 (20.7) |  | At 24mo: 13.6 (19.8) |
|  |  |  |  | *Mean (SD); g/kg/day* | | *Mean (SD); g/kg/day* | | *Mean (SD); g/kg/day* | |
|  |  |  |  | 1.6 (0.3) | At 3mo: 1.6 (0.2) | 0.4 (0.1) | At 3mo: 1.3 (0.5) | 1.2 (0.2) | At 3mo: 0.3 (0.5) |
|  |  |  |  |  | At 24mo: 1.5 (0.5) |  | At 24mo: 1.1 (0.4) |  | At 24mo: 0.4 (0.6) |
|  |  |  |  | *Median (range); g/day* | | *Median (range); g/day* | | *Median (range); g/day* | |
|  |  |  |  | 47.7 (31.0-63.0) | At 3mo: 52.4 (28.3-87.3) | 13.5 (7.0-19.0) | At 3mo: 38.4 (16.0-87.0) | 34.5 (21.3-48.0) | At 3mo: 0.0 (0.0-42.0) |
|  |  |  |  |  | At 24mo: 56.7 (41.0-76.0) |  | At 24mo: 44.5 (19.0-76.0) |  | At 24mo: 5.4 (0.0-57.0) |
|  |  |  |  | *Median (range); g/kg/day* | | *Median (range); g/kg/day* | | *Median (range); g/kg/day* | |
|  |  |  |  | 1.6 (1.1-2.0) | At 3mo: 1.6 (1.3-1.9) | 0.4 (0.3-0.8) | At 3mo: 1.4 (0.5-1.9) | 1.2 (0.8-1.5) | At 3mo: 0.0 (0.0-1.4) |
|  |  |  |  |  | At 24mo: 1.5 (0.8-2.3) |  | At 24mo: 1.1 (0.6-1.5) |  | At 24mo: 0.1 (0.0-1.7) |
| Ünal 2015  [50]  Gökmen Özel 2014  [52] | *Mean (SD, range)* | *Mean (SD); mg/day* | | *Mean (SD); g/day* | | n/a | | *Mean (SD); g/day* | |
|  | 30.2 (11.1, 6-48.6) | 655.6 (284.1) | 2382.0 (1043.0) | 31.1 (9.7) | 55.4 (21.9) |  | | 13.4 (6.8) | 1.4 (3.8) |
|  |  | *Mean (SD); mg/kg/day* | | *Mean (SD); g/kg/day* | |  | | *Mean (SD); g/kg/day* | |
|  |  | 37.4 (15.0) | 97.3 (33.7) | 1.7 (0.4) | 2.3 (0.7) |  | | 0.7 (0.3) | 0.1 (0.3) |
|  |  | *Median (range); mg/day* | | *Median (range); g/day* | |  | | *Median (range); g/day* | |
|  |  | 600.0 (249.0-1680.0) | 2240.0 (302.0-5013.0) | 31 (7.6-50.4) | 50.8 (7-108.7) |  | | 13.4 (1.3-35.0) | 0.0 (0.0-16.7) |
|  |  | *Median (range); mg/kg/day* | | *Median (range); g/kg/d* | |  | | *Median (range); g/kg/day* | |
|  |  | 35.0 (9.0-80.0) | 97.0 (30.0-184.0) | 2.0 (0.9-2.4) | 2.3 (0.7-4.0) |  | | 0.7 (0.1-1.2) | 0.0 (0.0-1.4) |
| Feldmann 2017 [16] | 6 | *Mean (range); mg/kg/day* | | *Mean (SD); g/day* | | n/a | | *Mean (SD); g/kg/day* | |
|  |  | 13.8 (4.3-42.4) | n/a | 18.5 (18.4) | 35.5 (14.1) |  | | 0.76 (0.34) | 0.39 (0.36) |
| Rocha 2017 [54] | *Mean (SD, range)* | *Mean (SD); mg/day* | | *Mean (SD); g/day* | | *Mean (SD); g/day* | | *Mean (SD); g/day* | |
|  | 12.3 (4.5, 3.0-17.0) | 1299.0 (583.0) | 2365.0 (766.0) | 70.7 (21.7) | 81.7 (16.7) | 28.7 (13.4) | 50.3 (16.5) | 41.3 (12.7) | 31.3 (10.5) |
|  |  | *Mean (SD); mg/kg/day* | | *Mean (SD); g/kg/day* | | *Mean (SD); g/kg/day* | | *Mean (SD); g/kg/day* | |
|  |  | 27.6 (8.3) | 46.4 (8.8) | 1.5 (0.3) | 1.7 (0.3) | 0.6 (0.2) | 1.0 (0.2) | 0.9 (0.2) | 0.7 (0.3) |
|  |  | *Median (range); mg/day* | | *Median (range); g/day* | | *Median (range); g/day* | | *Median (range); g/day* | |
|  |  | 1122.0 (644.0-2414.0) | 2414.0 (1439.0-3740.0) | 68.4 (44.4-107.6) | 81.4 (57.2-102.2) | 24.2 (14.0-52.6) | 52.6 (29.7-79.1) | 40.0 (22.8-61.0) | 36.0 (10.0-41.0) |
|  |  | *Median (range); mg/kg/day* | | *Median (range); g/kg/day* | | *Median (range); g/kg/day* | | *Median (range); g/kg/day* | |
|  |  | 28.6 (128-42.4) | 47.1 (32.3-64.7) | 1.7 (0.9-1.9) | 1.7 (1.2-2.1) | 0.6 (0.3-0.9) | 1.0 (0.7-1.3) | 1.0 (0.5-1.1) | 0.8 (0.2-1.0) |
| Evers 2018 [44]^e^ | 54.0-66.0 | n/a | | *Mean (SD); g/kg/day* | | *Mean (SD); g/kg/day* | | *Mean (SD); g/kg/day* | |
|  |  |  | | 1.5 (0.5) | 1.0 (0.2) | 0.4 (0.2) | 0.7 (0.3) | 1.1 (0.5) | 0.3 (0.3) |
|  |  |  | | *Median (range); g/kg/day* | | *Median (range); g/kg/day* | | *Median (range); g/kg/day* | |
|  |  |  | | 1.4 (0.4-2.4) | 0.9 (0.8-1.4) | 0.4 (0.2-0.9) | 0.8 (0.2-1.2) | 1.0 (0.2-1.9) | 0.4 (0.0-1.0) |
| Paras 2018  [53] | ≥3.0 | *Mean (SD); mg/day* | | n/a | | n/a | | *Mean (SD); g/day* | |
|  |  | 1065.4 (492.2) | n/a |  | |  | | n/a | 0.0 (0.0) |
|  |  | *Median (range); mg/kg/day* | |  | |  | |  |  |
|  |  | 1250.0 (325.0-1750.0) | n/a |  | |  | |  |  |

Abbreviations: BH4: tetrahydrobiopterin; IQR: interquartile range; mo: months; Phe: phenylalanine; PS: protein substitute; SD: standard deviation; n/a: not available.

^a^ Singh 2010: only self-reported intakes are shown in the table. Mean prescribed Phe intakes before BH4 and at 3 months, 1 year and 2 years of follow-up were 11.9±4.1 mg/kg/day, 39.9±11.5 mg/kg/day, 40.0±12 mg/kg/day and 37.0±9.0 mg/kg/day, respectively.

^b^ Singh 2011: Data shown as means (SD) are prescribed Phe/protein substitute intakes. One patient was lost to follow-up between 4 months and 1 year (n=18 and 17, respectively) and mean (SD) at baseline was re-calculated for the 17 patients with 1 year follow-up. Data shown as median (IQR) are self-reported dietary intakes at 1 year follow-up.

^c^ Leuret 2012: Only 8/15 patients were on a Phe-restricted diet before BH4 and were therefore included; however, duration of BH4 treatment was only available for the total sample of 15 patients.

^d^ Aldámiz-Echevarría 2013: Unclear if patients with a 5y follow-up were also described in the group of patients with a 2y follow-up. It was assumed that the 2 cohorts comprised different patients.

^e^ Evers 2018: Only data on prescribed intakes were available in the original article.
