# Supplementary figures and images for: Protein Substitute Requirements of Patients with Phenylketonuria on BH4 Treatment: A Systematic Review and Meta-Analysis
Source: Nutrients. 2021 Mar 23;13(3):1040. doi: 10.3390/nu13031040 (PMC8004763; doi:10.3390/nu13031040)

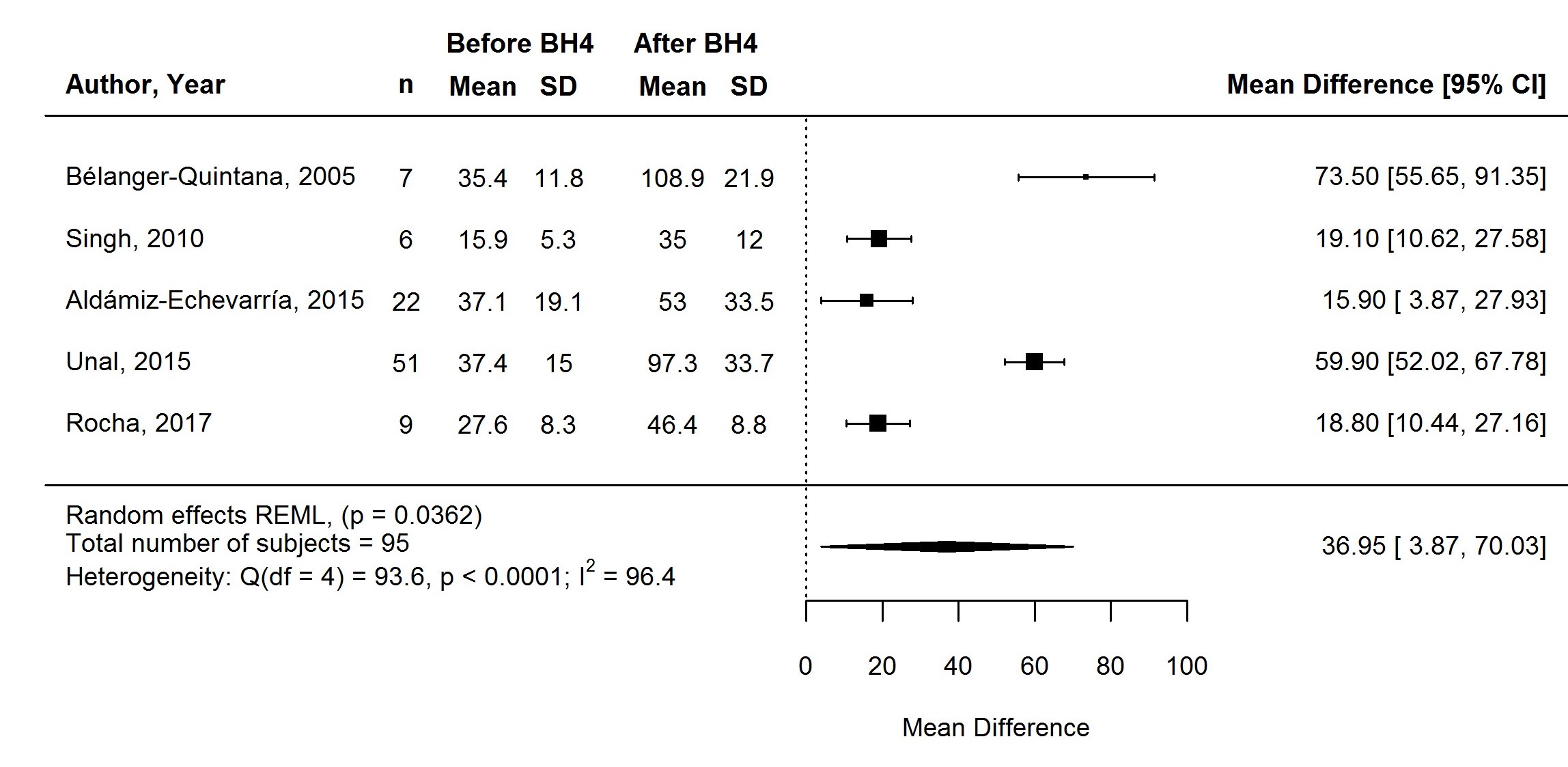

Supplement: Supplementary file 1 [file nutrients-13-01040-s001.zip › SupplementaryFiles/SupplMat Fig S1. Phe intake mgkgday.jpg]

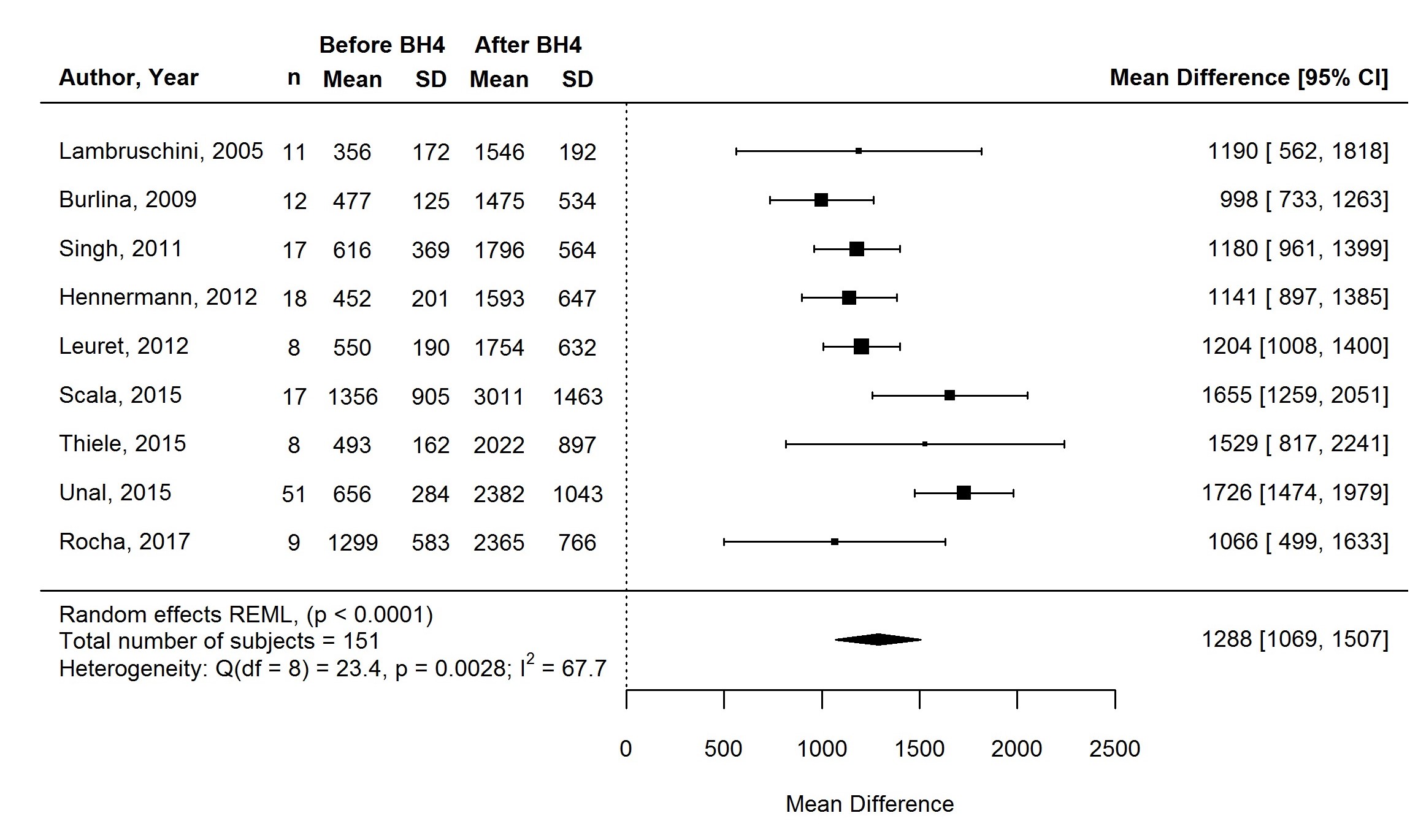

Supplement: Supplementary file 1 [file nutrients-13-01040-s001.zip › SupplementaryFiles/SupplMat Fig S2. Phe intake mgday.jpg]

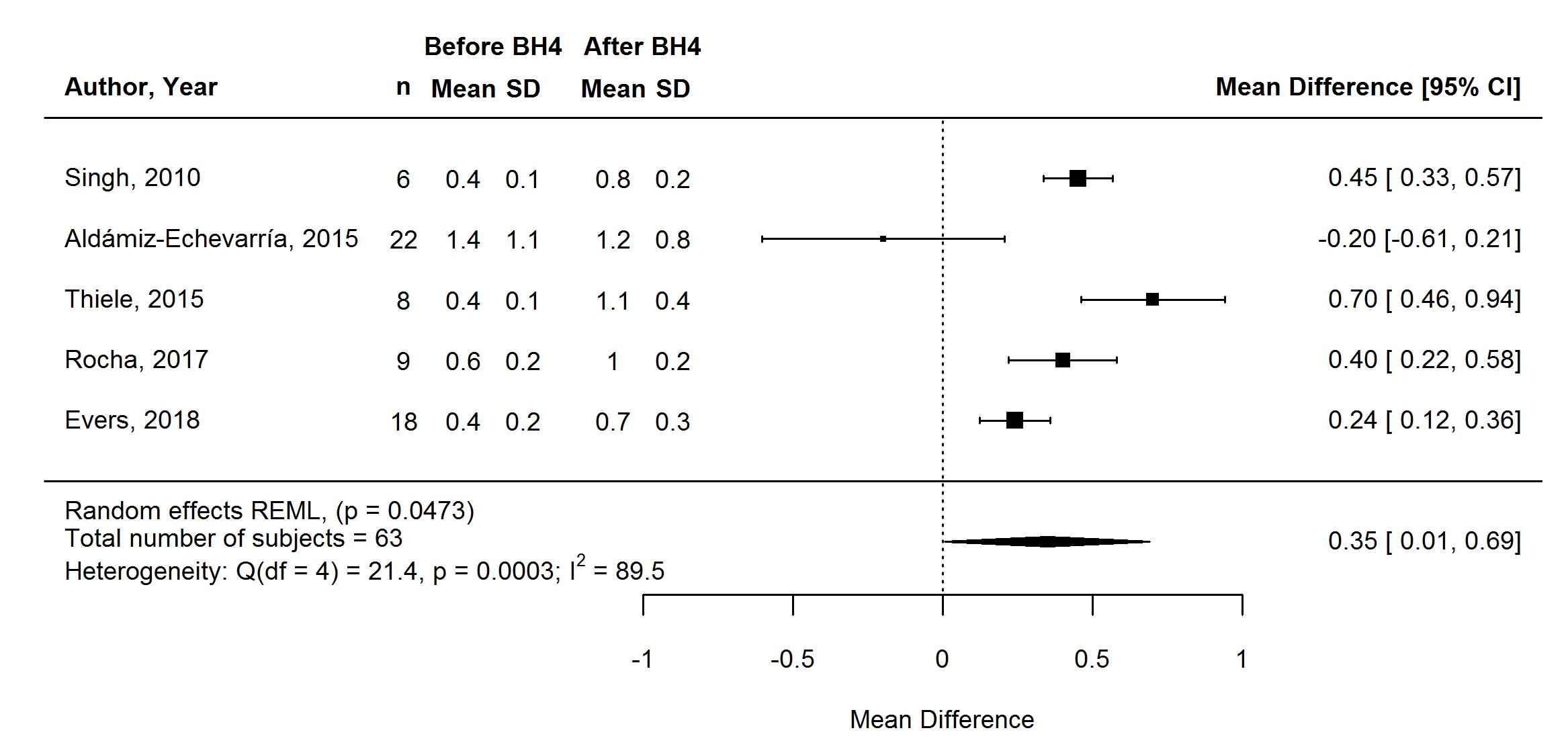

Supplement: Supplementary file 1 [file nutrients-13-01040-s001.zip › SupplementaryFiles/SupplMat Fig S3. Natural protein gkgday.jpg]

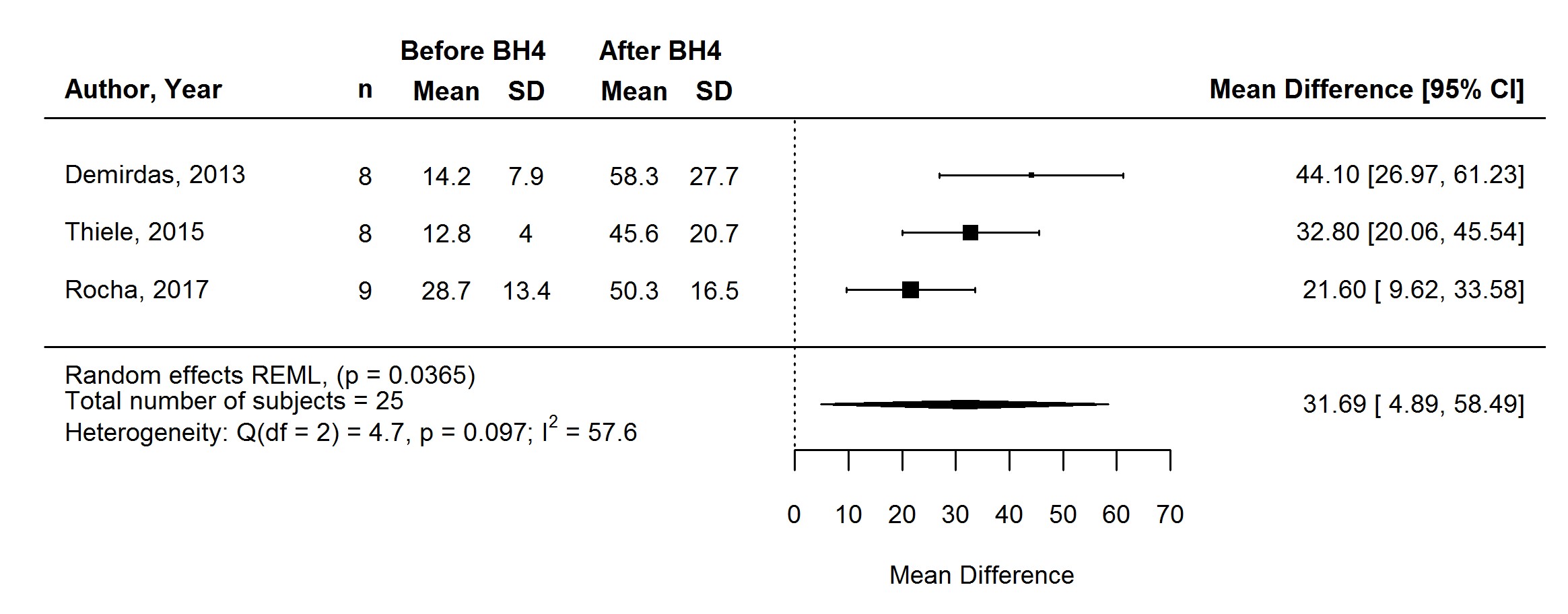

Supplement: Supplementary file 1 [file nutrients-13-01040-s001.zip › SupplementaryFiles/SupplMat Fig S4. Natural protein gday.jpg]

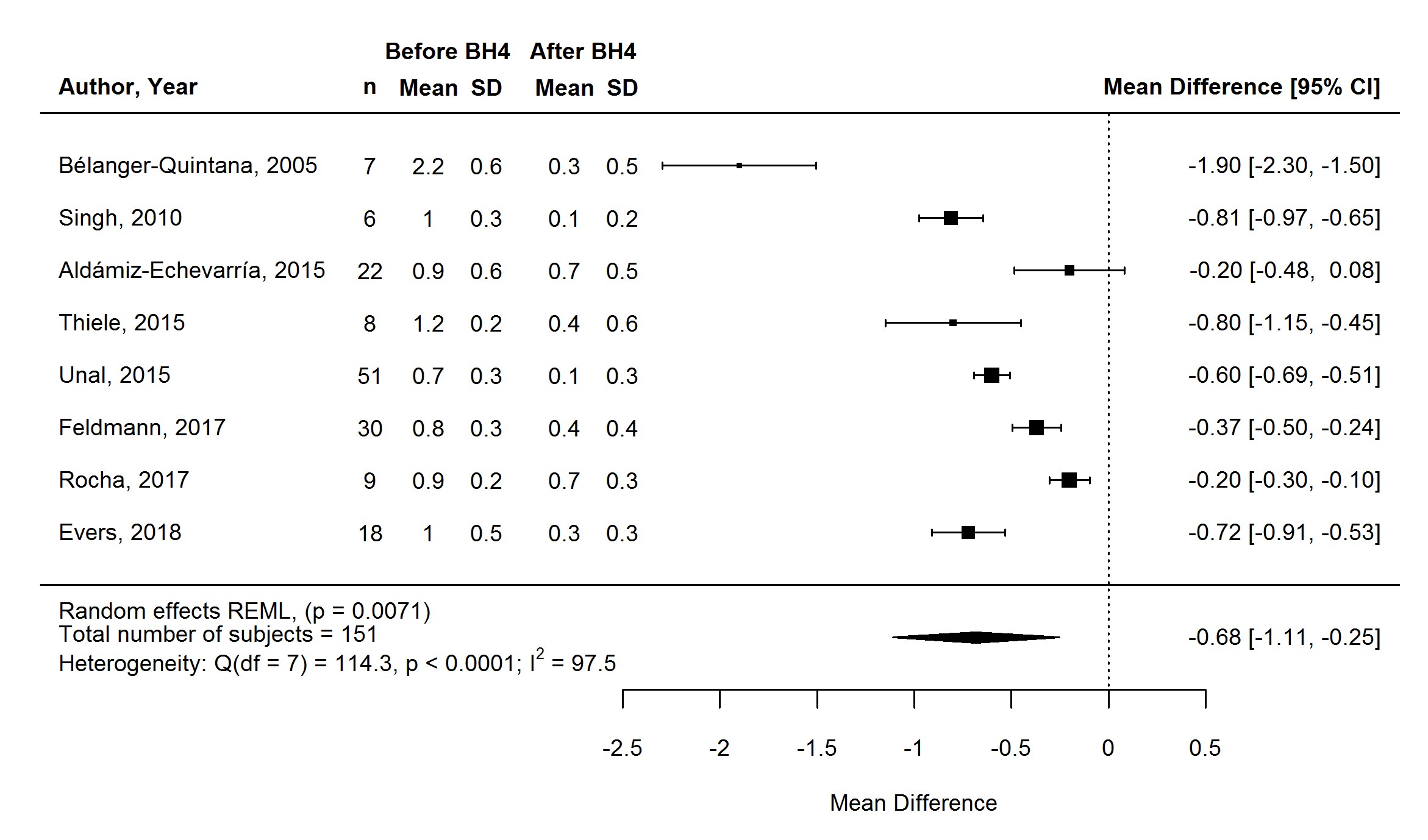

Supplement: Supplementary file 1 [file nutrients-13-01040-s001.zip › SupplementaryFiles/SupplMat Fig S5. PS gkgday.jpg]

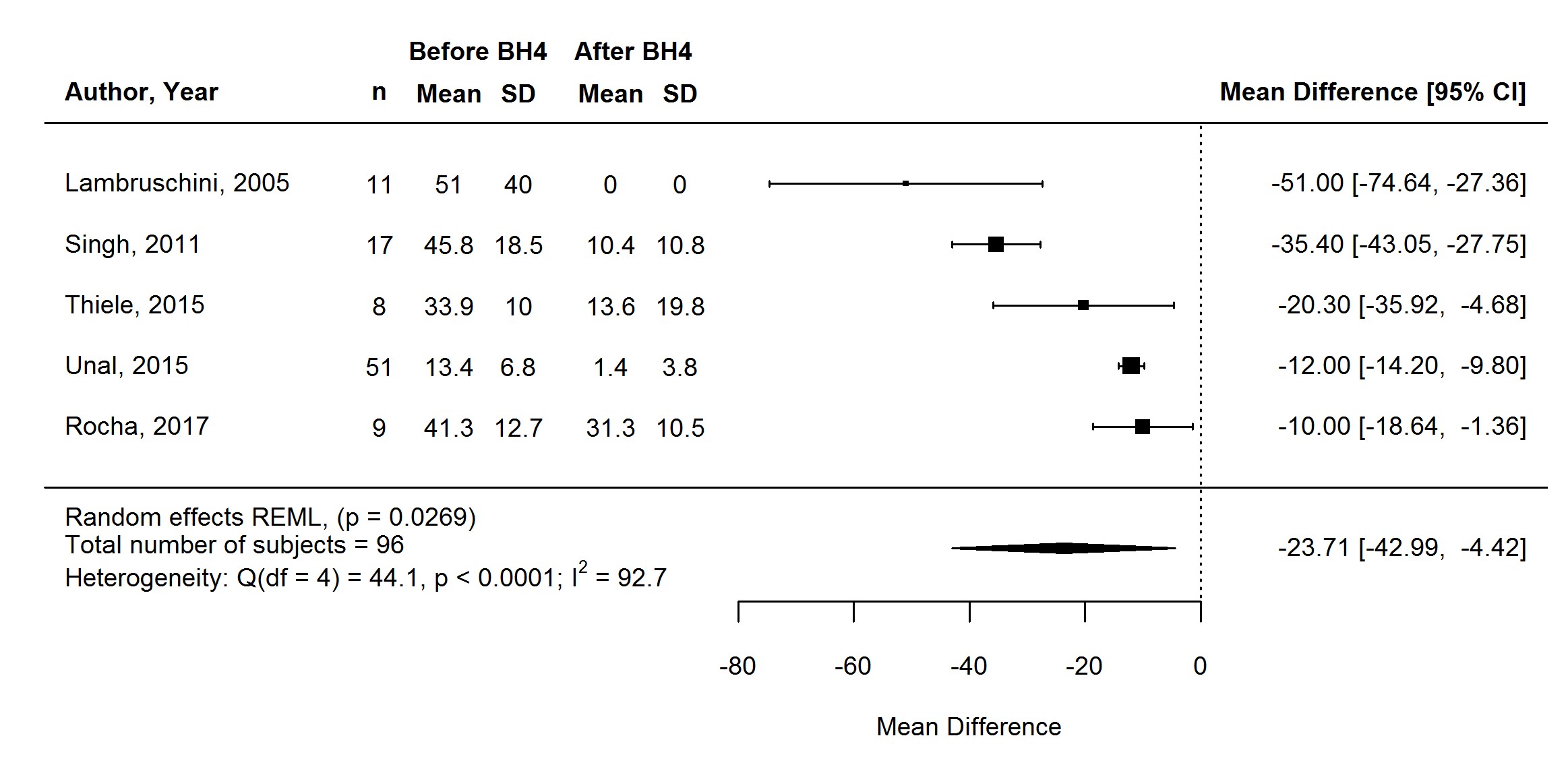

Supplement: Supplementary file 1 [file nutrients-13-01040-s001.zip › SupplementaryFiles/SupplMat Fig S6. PS gday.jpg]

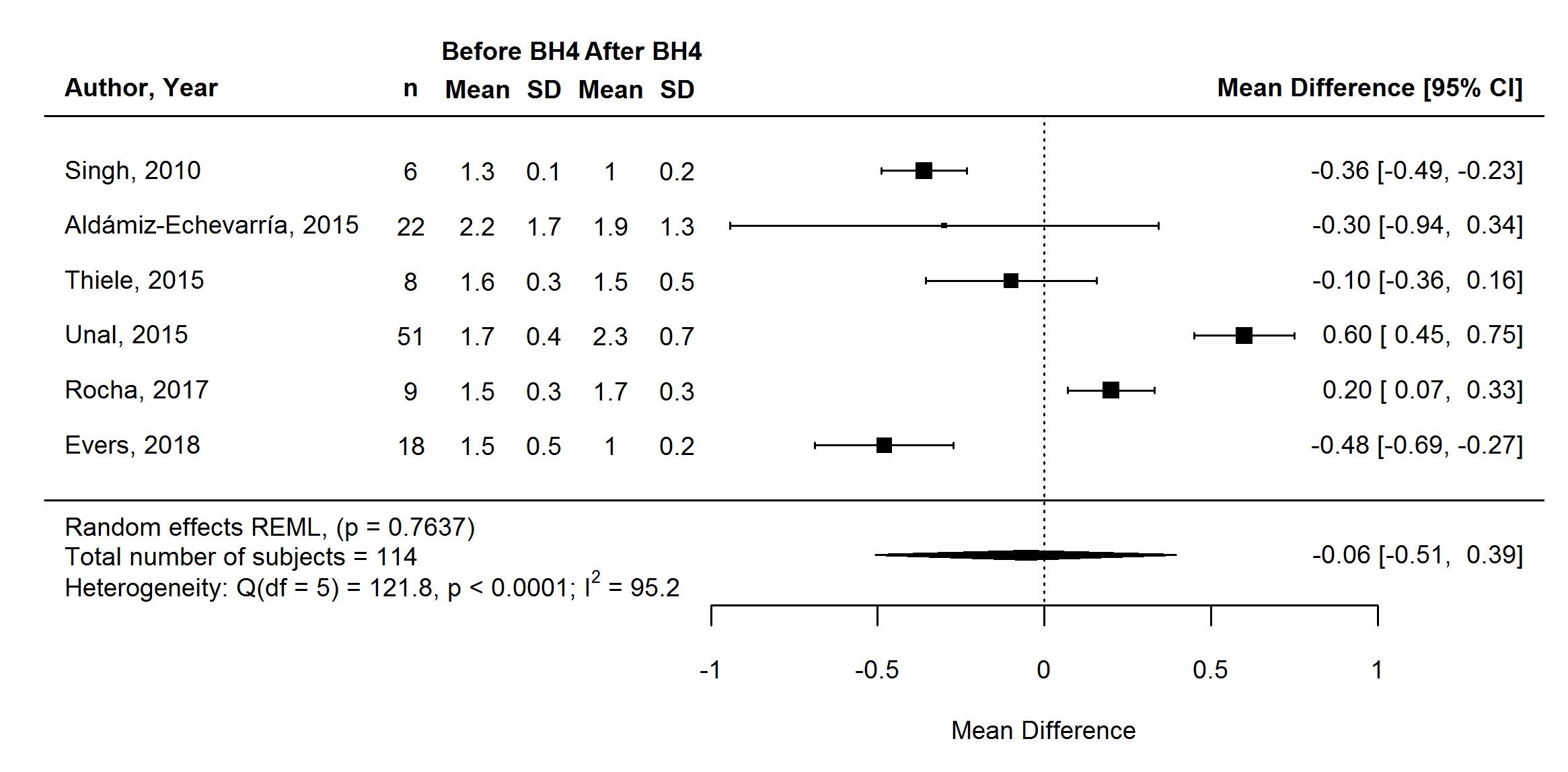

Supplement: Supplementary file 1 [file nutrients-13-01040-s001.zip › SupplementaryFiles/SupplMat Fig S7. Total protein gkgday.jpg]

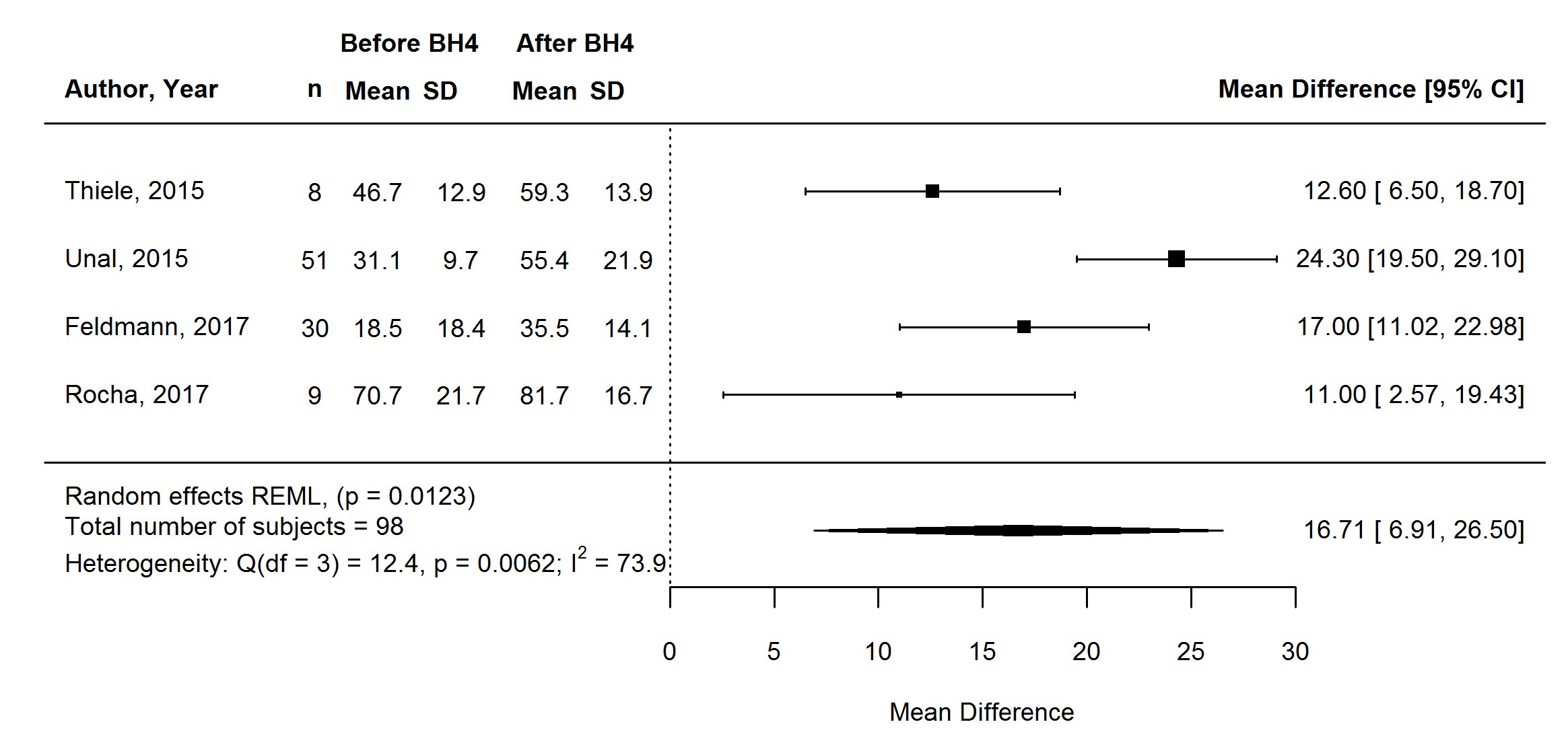

Supplement: Supplementary file 1 [file nutrients-13-01040-s001.zip › SupplementaryFiles/SupplMat Fig S8. Total protein gday.jpg]
